# Allele-specific DNA demethylation editing leads to stable upregulation of allele-specific gene expression

**DOI:** 10.1016/j.isci.2024.111007

**Published:** 2024-09-23

**Authors:** Nivethika Rajaram, Katharina Benzler, Pavel Bashtrykov, Albert Jeltsch

**Affiliations:** 1 Institute of Biochemistry and Technical Biochemistry, University of Stuttgart, Allmandring 31, 70569 Stuttgart, Germany

**Keywords:** Biological sciences, Biochemistry, Biochemistry Applications

## Abstract

Epigenome editing is an emerging technology that allows to rewrite epigenome states and reprogram gene expression. Here, we have developed allele-specific DNA demethylation editing at gene promoters containing an SNP by sgRNA/dCas9 mediated recuitment of TET1. Maximal DNA demethylation (up to 90%) was observed 6 days after transient transfection of the epigenome editors and it was almost stable for 15 days. After allele-specific targeting, DNA demethylation was up to 15-fold more efficient at the targeted allele. Our data show that locus-specific and allele-specific DNA demethylation can trigger the expression of previously silenced genes. Allele-specific DNA demethylation shifted allelic expression ratios about 4-fold. Allele-specific DNA demethylation could be used to correct aberrant imprinting in patients suffering from imprinting disorders and to study the roles of individual alleles of a gene in a given cellular context.

## Highlights

• sgRNA/dCas9 targeting of TET1 was used for allele-specific DNA demethylation (ASD)
• Allele discrimination was based on an SNP in the PAM site of dCas9
• ASD led to strong and specific demethylation of one allele of the target locus
• Demethylation was stable over several days and led to allele-specific gene expression

## Graphical abstract

**Figure:**
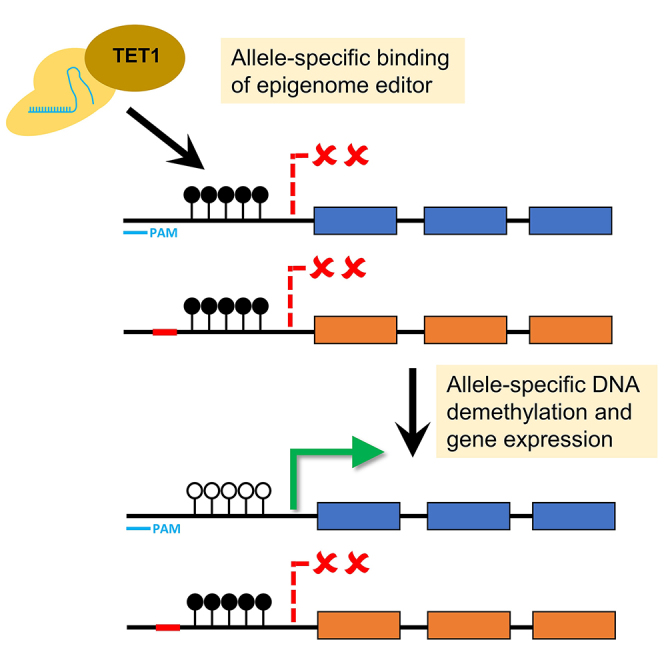

## Introduction

DNA methylation and histone post-translational modifications regulate chromatin states in human cells.^1^ These epigenomic marks are set, removed, and read by numerous chromatin-modifying and interacting complexes which altogether control gene activity, embryonic development, cellular differentiation, and adaptation of cells to environmental conditions.^2^^,^^3^^,^^4^ DNA methylation is introduced by DNA methyltransferases^5^ and actively removed by the ten-eleven translocation (TET) protein family of dioxygenases catalyzing the conversion of 5-methylcytosine (5mC) to its oxidized derivatives, 5-hydroxymethylcytosine, 5-formylcytosine, and 5-carboxylcytosine,^6^^,^^7^^,^^8^^,^^9^ which is followed by the removal of the higher oxidized forms catalyzed by thymine-DNA glycosylase.^10^

Epigenome editing refers to the locus-specific rewriting of the epigenome modifications using so called epigenome editors (EpiEditors) with the aim to reprogram chromatin states and gene expression.^11^^,^^12^^,^^13^^,^^14^^,^^15^ For DNA methylation editing, the catalytic domains of DNMT3A (or DNMT3A and DNMT3L)^16^^,^^17^^,^^18^^,^^19^^,^^20^^,^^21^^,^^22^ have been recruited to selected target regions through a nuclease-deactivated CRISPR-associated protein 9 (dCas9)^23^ complexed with a specific single guide RNA (sgRNA).^15^ Similar experiments have been conducted for locus-specific DNA demethylation using the catalytic domain TET1 (TET1CD).^16^^,^^21^^,^^24^^,^^25^^,^^26^^,^^27^ In both systems, the sgRNA recognizes the target DNA sequence by forming Watson-Crick base pairs with one of the DNA strands generating an RNA-DNA hybrid at the target site. In addition, the dCas9 protein directly interacts with the DNA at a trinucleotide proto-spacer adjacent motif (PAM) which is located directly at the 3′ end of the target DNA sequence and in the case of dCas9 has the sequence NGG (where N stands for any nucleotide). The dCas9 protein can be fused to a SunTag repeat sequence^28^ to recruit multiple effector domains fused to single-chain variable fragment (scFv) that specifically binds the SunTag. This system was very efficient and showed high specificity in DNA demethylation using TET1CD as effector domain.^24^^,^^27^

Recently, a more specific variant of epigenome editing was introduced in which DNA methylation was selectively introduced in only one allele of the target locus, which was defined by an SNP in the sgRNA binding site or PAM region of dCas9 (allele-specific methylation, ASM).^22^ Here, we aimed to complement this approach by introducing allele-specific DNA demethylation (ASD). For this, we identified target genes with methylated promoters and an SNP in the promoter region in HEK293 cells and selectively targeted the TET1CD domain to only one defined allele of the target locus. We explored ASD with respect to locus and allele-specificity, stability and its effects on allele-specific gene expression. Our data show that strong and specific ASD could be achieved in three test cases, which was stable over 15 days and led to an upregulation of the expression of the demethylated allele. These results indicate that ASD can be applied in future experiments aiming toward the selective activation of one specific allele of a gene of interest.

## Results

### Target selection in the HEK293 cell line

**Figure 1:**
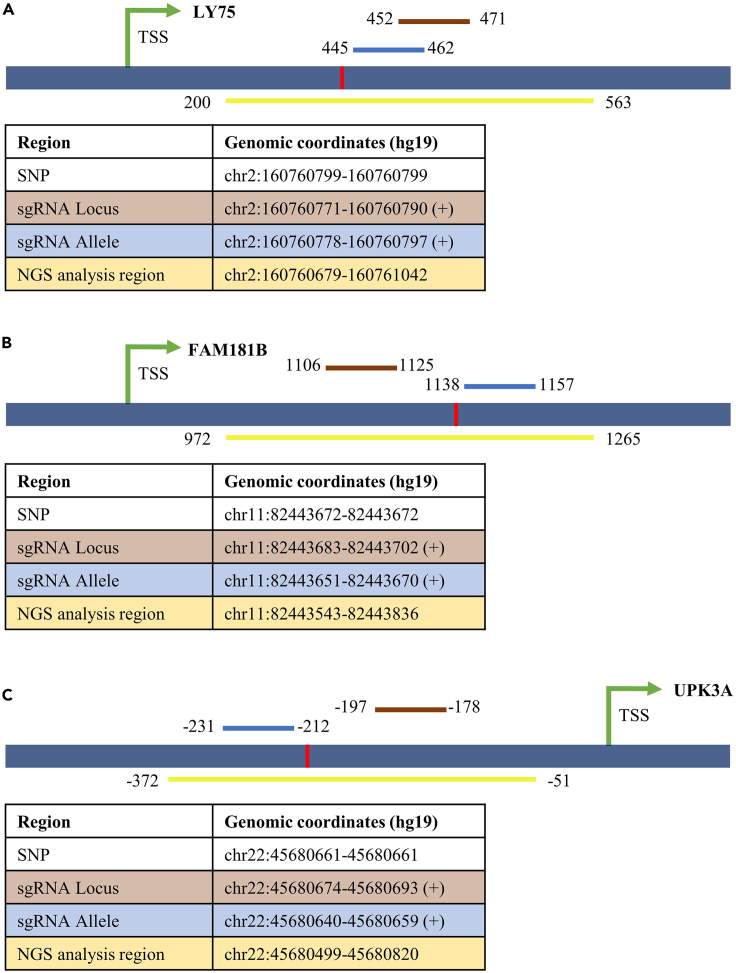
Schematic images of the genomic target loci (A) LY75 locus. (B) FAM181B locus. (C) UPK3A locus. Shown are the positions of the locus-specific sgRNA binding site (brown bar), the allele-specific sgRNA binding site (blue bar), the genomic region analyzed by NGS (yellow bar) and the TSS of the associated gene starting at position +1 (green arrow). The numbers indicate the distance in bps from the TSS. Some of the features are not drawn to scale. In the tables, the genomic co-ordinates of the corresponding positions are provided. The positions of the SNPs are indicated by red lines. See also Figures S1 and S2.

The HEK293 cell line was used as the model cell line for development and first application of ASD. The genomic sequence and SNP information of HEK293 were obtained from a public database.^29^ A list of methylated CpG islands (CGIs) of HEK293 was obtained from MBD-sequencing data (NCBI GEO accession number GSE144331).^20^ Promoter regions containing methylated CGIs (here defined as ±2000 bp surrounding the TSS) were chosen as suitable targets for locus-specific DNA demethylation because of high chances to affect gene transcription. Among them, the regions containing a G-to-Y SNP in an NGG context that can be employed as PAM site to enable discrimination of the alleles were prioritized.^30^ Using this approach, the promoters of the *LY75*, *FAM181B*, and *UPK3A* genes were finally selected for ASD. For these regions, conventional sgRNAs for locus-specific binding and special sgRNAs for allele-specific targeting were designed with same directionality and binding very close to each other (+/− 20 bp apart from each other). The selected target loci are schematically shown in Figure 1 and details of the design of the locus-specific and allele-specific sgRNA and the corresponding regions for DNA methylation analysis are provided in Figures S1 and S2.

### Locus-specific demethylation of the genomic target regions

The promoter regions of the *LY75*, *FAM181B*, and *UPK3A* genes were first targeted at the locus-specific level. Two plasmids were used for EpiEditing, one encoding dCas9-5X SunTag together with scFv-TET1CD and GFP, and the multi-sgRNA vector expressing the locus-specific LY75, UPK3A, and FAM181B sgRNAs and dsRed as second one. In the different experiments reported here, epigenome editing was achieved using the general workflow provided in Figure S3. First, the EpiEditing constructs were transiently co-transfected into the HEK293 cells. As a control, a plasmid expressing a scrambled sgRNA not having a target site in the human genome^20^^,^^31^ was also used together with the EpiEditing construct. Then, the cells positive for both vectors were enriched by fluorescence activated cell sorting (FACS) on day 3 post transfection and the cells were maintained in the culture until day 15. Genomic DNA and RNA samples were collected at day 3, 6, 9, and 15. Afterward, DNA methylation analysis of the target regions was performed by amplicon-based bisulfite sequencing.^30^ Finally, the obtained methylation data were processed and the methylation profiles that represent the methylation levels at each CpG site were calculated.

**Figure 2:**
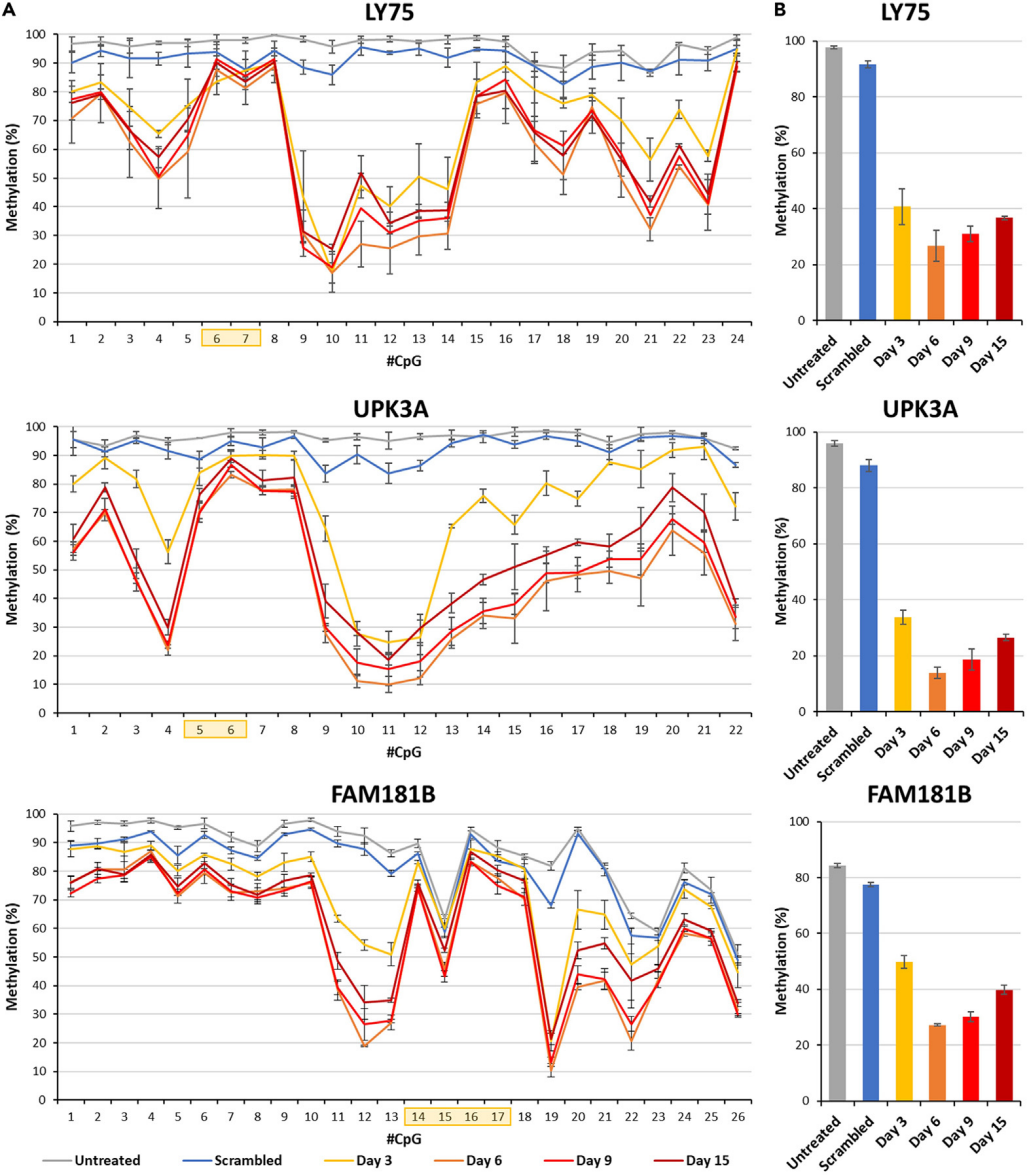
Locus-specific demethylation of the targets (A) Methylation profiles of the locus-specific demethylation of the target LY75, FAM181B and UPK3A. The x axis and the y axis represent the CpG sites in the region included for the NGS analysis and the methylation levels, respectively. The sgRNA binding sites are indicated by yellow shaded boxes. The cells treated with scrambled sgRNA and EpiEditing construct were collected on day 6. The cells treated with respective sgRNA and the EpiEditing constructs were collected at different time points (day 3, 6, 9, and 15) and labeled correspondingly. (B) Quantitative analysis of the methylation at each locus across different time points. The CpG sites selected for quantitate analysis of LY75 were 9, 10, 11, 12, 13, and 14. For FAM181B the sites 11, 13, 19, 20, and 22 were used and in the case of UPK3A the sties 4, 10, 11, and 12. The experiments in panel (A) and (B) were conducted in biological triplicates. The lines and bars show the average, error bars display the SD.

In the case of LY75, the sgRNA/dCas9 complex binds to the methylated promoter region at the CpG sites 6 and 7 of the analyzed region. As shown in Figure 2A, the LY75 region showed highest methylation in the untreated samples. The treated samples collected on day 3 exhibited a considerable decline of methylation levels, but the highest demethylation of the targeted region was observed on day 6. The samples collected on day 9 and day 15 exhibited a gradual increase in the methylation levels compared to day 6 but did not reach methylation levels of the untreated sample. Moreover, the footprint of the sgRNA/dCas9 complex is clearly visible in the demethylation profile of the treated samples, because strong demethylation is observed at several CpG sites, but the sites 6 and 7 were protected from the activity of the TET1CD enzyme.

For quantitative analysis, the CpG sites with at least 50% of the highest demethylation effect were selected, in case of LY75 the CpG sites from 9 to 14 (Figure 2B). For these selected sites, the original methylation (97.7%) dropped to 26.8% on day 6 corresponding to a level of demethylation of 73% compared to the parental methylation. On day 15, the methylation level was 36.7% corresponding to 62.4% demethylation and indicating that 86% of the initial demethylation remained stable. The sample treated with scrambled EpiEditors was analyzed at day 6 and exhibited a minimal reduction of methylation (about 12%) indicating that specific editing was about 6-times more efficient than the background activity of the untargeted epigenome editing complex.

In case of UPK3A, the sgRNA/dCas9 complex bound to the CpG sites 5 and 6 (Figure 2A). The CpG sites selected for the quantitative analysis were 4, 10, 11, and 12. In this case, 86% and 72% demethylation were observed at day 6 and day 15 (Figure 2B). About 11% demethylation was observed with the scrambled sgRNA, indicating that specific editing was about 8-fold more efficient than background activity. At the FAM181B target, the sgRNA/dCas9 complex bound to the CpG sites 14, 15, 16, and 17. As shown in Figure 2, strong demethylation was observed at many CpG sites, but the sgRNA/dCas9 binding site remained methylated. The CpG sites 11, 13, 19, 20, and 22 were selected for the quantitative analysis. For these sites, 68% demethylation was observed at day 6 and at day 15, 78% of the initial demethylation was still observed.

In summary, the locus-specific demethylation effects of all three targets were similar: strong and specific demethylation was observed at day 6 which remained partially stable until day 15, when a large fraction of the initial demethylation was still observed. All these experiments were conducted as biological triplicates.

### Specificity of the locus-specific DNA demethylation

The specificity of a locus-specific demethylation can be compromised by TET1CD activity in untargeted genomic regions caused either by the activity of the untargeted effector domain or by binding of the sgRNA/dCas9 complex at an off-target site, which then recruits the TET1CD to the off-target site. Off-target site binding of sgRNA/dCas9 complexes typically occurs at genomic regions with sequence similarity to the sgRNA target site. The first mode of off-target activity can be detected by using scrambled sgRNA that does not have a binding site in the human genome.^20^^,^^31^ Additionally, the off-target demethylation caused by the untargeted activity of the effector domain can be investigated at unrelated methylated genomic regions that are not bound by any of the sgRNA/dCas9 complexes. In this work, the SLC6A3 and MEST loci, both methylated in HEK293 cells, were used for that purpose. The demethylation of the unrelated off-target regions was analyzed in untreated samples, in samples treated with scrambled sgRNA and in samples treated with target specific sgRNA. As described previously and shown in Figure 2, the samples treated with EpiEditors and scrambled sgRNA were analyzed on day 6. The samples treated with EpiEditors and gene specific sgRNA were analyzed on day 6 and day 15 (Figure S4) and showed a weak global demethylation of <10–12%.

The off-target binding of the sgRNA/dCas9 complex is typically observed at genomic regions with a small number of mismatches to the sgRNA used for targeting. These potential off-target regions can be identified using the online platforms https://cctop.cos.uni-heidelberg.de/ ^32^ and http://www.rgenome.net/cas-offinder/.^33^ Among the list of tentative off-targets for each sgRNA, the regions with methylated CpG sites in HEK293 cells were selected for further examination (Table S1). The methylation levels of the selected regions were analyzed in the untreated samples, samples treated with scrambled EpiEditors (day 6) and the samples treated with target-specific EpiEditors (day 6 and day 15). As shown in Figure S5, depending on the target site, demethylation levels of 3–15% were observed. Comparison of the demethylation levels after treatment with scrambled and locus-specific sgRNA indicates that in each case about 70–80% of the off-target demethylation was caused by an untargeted activity of the TET1CD and only a minor part was due to off-target binding of the sgRNA/dCas9 complex. Overall, these data indicate a good specificity of the locus-specific DNA demethylation editing at both loci.

### Allele-specific demethylation of the target loci

To target the LY75, FAM181B, and UPK3A promoters at allelic level, sgRNAs were designed to bind one specific allele in each case. The target regions were selected to contain a heterozygous point mutation (G>C) which is flanked by another guanine. The allele containing the GG dinucleotide consequently carries a PAM site and represents the target allele for demethylation. The 20 nt sequence upstream of the selected PAM was used to design the corresponding allele-specific sgRNA (see Figure S1). It has to be noted that the SNPs in the target alleles can change the number and exact location of the CpG sites. The designed sgRNAs for LY75, FAM181B, and UPK3A were cloned in a multi-sgRNA expression vector. The experimental workflow including the DNA methylation analyses was conducted as described for the locus-specific DNA demethylation editing (Figure S3), except that the bisulfite sequencing data were analyzed with allelic resolution.

**Figure 3:**
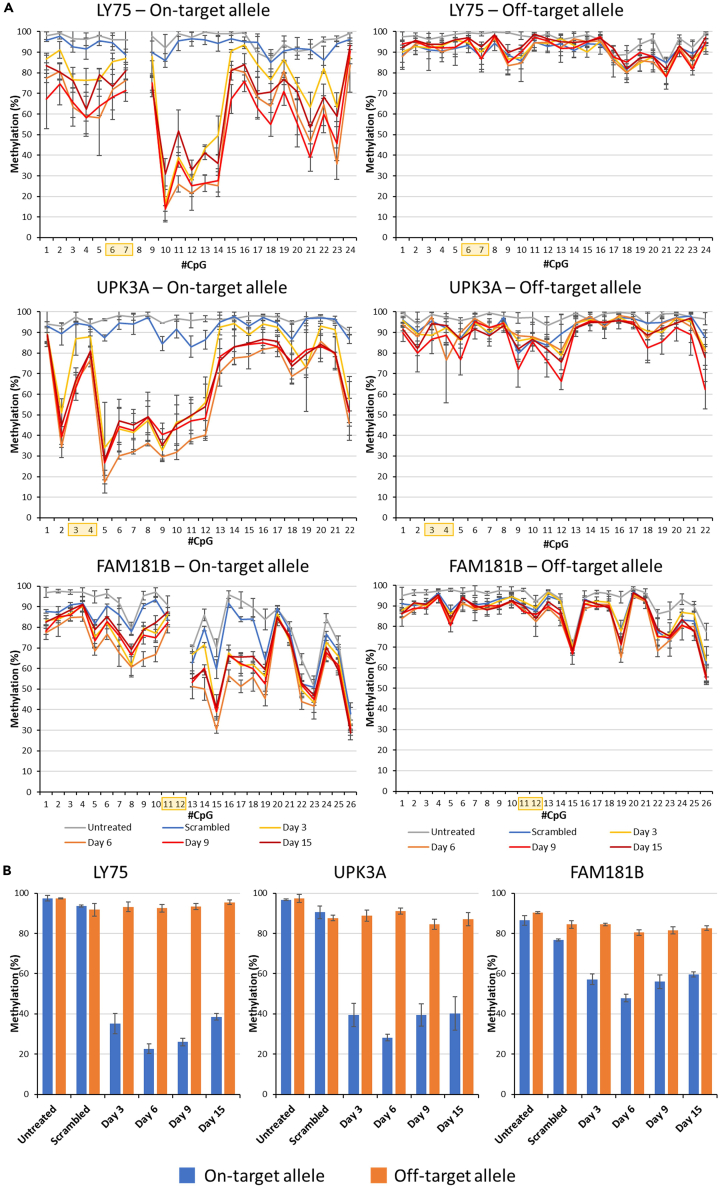
Allele-specific demethylation of the targets (A) Methylation profile of the on-target and off-target alleles for the targets LY75, UPK3A and FAM181B. The x axis and the y axis represent the CpG sites in the region included for the NGS analysis and the methylation levels, respectively. The sgRNA binding sites are indicated by yellow shaded boxes. The cells treated with scrambled sgRNA and EpiEditing construct were collected on day 6. The cells treated with allele-specific sgRNA and the EpiEditing constructs were collected at different time points (day 3, 6, 9, and 15) and labeled correspondingly. (B) Quantitative analysis of the methylation at each locus across different time points. The CpG sites selected for quantitate analysis of LY75 were 10, 11, 12, 13, and 14. In case of FAM181B sites 15, 16, 17, 18, and 19 were used in for UPK3A 5, 6, 7, 9, and 10. In case of UPK3A due to the SNP, the annotated CpG sites differ between both alleles. The experiments in panel (A) and (B) were conducted in biological triplicates. The lines and bars show the average, error bars display the SD.

In allele-specific targeting, the targeted allele is referred as the “on-target allele” and the untargeted allele is referred as “off-target allele”. In case of LY75, the sgRNA/dCas9 complex binds to the CpG sites 6 and 7 in the analyzed region. The on-target allele of the untreated samples exhibited high methylation levels (Figure 3). The on-target allele of the sample treated with scrambled sgRNA showed a <14% reduction in methylation. The on-target allele of the sample treated with the specific sgRNA collected on day 3 exhibited a strong loss of DNA methylation compared to the untreated samples. The highest demethylation effects were observed in the on-target allele of the treated samples collected on day 6. The CpG sites 10, 11, 12, 13, and 14 were selected for quantitative analysis and showed an overall 77% reduction of methylation. The on-target allele of the samples collected on day 9 and 15 showed a slight gradual increase in the methylation levels compared to their counterpart collected on day 6, but methylation levels did not reach the initial levels and 69% of the initial demethylation remained stable up to day 15.

The off-target allele of LY75 from the untreated sample showed high methylation levels in the range of 80–90% methylation across CpG sites. For quantitative analysis the same CpG sites as used for the on-target allele were used. The methylation levels of the off-target allele from the scramble sgRNA and allele-specific sgRNA treated samples both collected on day 6 exhibited very similar methylation profiles and very weak demethylation effects (∼2–5%). The off-target allele of the samples treated with allele-specific EpiEditors collected on days 9, and 15 notably exhibited methylation levels similar to the samples treated with scrambled EpiEditors. Overall, on-target allele epigenome editing was 15-fold more efficient than editing at the off-target allele. The off-target activity observed here must be due to the untargeted activity of the TET1CD as the SNP in the PAM site likely prohibits dCas9 binding.

The sgRNA/dCas9 complex specific for the target UPK3A bound to the CpG sites 3 and 4. The CpG sites 5, 6, 7, 9, and 10 were selected for quantitative analysis (Figure 3). The on-target allele showed 71% demethylation, 83% of which remained stable until day 15. In contrast, the off-target allele showed only 6% of demethylation indicating that on-target editing was 11-fold more efficient than off-target demethylation. The sgRNA/dCas9 complex specific for the target FAM181B bound to the CpG sites 11 and 12. The CpG sites 15, 16, 17, 18, and 19 were selected for quantitative analysis revealing 45% on-target and 11% off-target demethylation, which was the least specific effect observed. As shown in Figure 3, the relatively high off-target demethylation was mainly caused by demethylation of CpG site 19, which was equally seen in samples treated with the allele-specific and scrambled sgRNA suggesting that it was due to an untargeted activity of the TET1CD which for unknown reasons showed an activity peak at this site. If CpG site 19 was disregarded, similar results as observed with LY75 and UPK3A were obtained, with off-target demethylation of 6% and an overall specificity for on-target editing of 8-fold. Of note, in all cases of allele-specific editing (as in the locus-specific editing described previously), the strongest effects were observed 6 days post transfection.

### Specificity of the allele-specific DNA demethylation

The data presented so far document a very good specificity of the ASD with strong methylation changes in the on-target allele and only minor effects in the off-target alleles which are due to untargeted activity of the TET1CD complex. To further validate this finding, global untargeted TET1CD activity was also investigated at the SLC6A3 and MEST loci as described above (Figure S4). The demethylation at both off-target regions was comparable to the effects observed with samples treated with scrambled-sgRNA EpiEditor. In both cases, about 10–12% demethylation was noticed on day 6. On day 15, a regain in methylation was observed. Moreover, potential off-target binding sites of the sgRNAs used for ASD were also identified and analyzed (Figure S6 and Table S1). While no off-target activity was observed at the PCDH19 locus, at SDF4 and TUBB2B some demethylation was detected (15 and 20%) but this was still considerably less than observed at the on-target alleles. The similarity of the effects observed with the allele-specific sgRNA and the scrambled sgRNA indicates that off-target activity was mainly due to the untargeted activity of the TET1CD complex.

### Allele-specific analysis of the results obtained in locus-specific DNA demethylation

In the previous sections, we observed an efficient locus-specific and allele-specific demethylation in the targets LY75, FAM181B, and UPK3A. The EpiEditors used for locus-specific and allele-specific demethylation differ in the sgRNA design such that locus-specific sgRNA does not discriminate between the alleles, whereas the allele-specific sgRNA does. Based on this, equal levels of DNA demethylation editing were expected in both alleles after treatment with the locus-specific sgRNA. To test this conjecture, we used the sequencing data from locus-specific demethylation and analyzed methylation levels on both alleles separately. Indeed, this analysis revealed a similar activity of the locus-specific epigenome editor on both alleles in all three cases (Figure S7). The variation of demethylation levels between the alleles were statistically insignificant (*p* value >0.05 determined by two-sided t-test assuming equal variance).

### Effect of demethylation on the expression of the target genes

**Figure 4:**
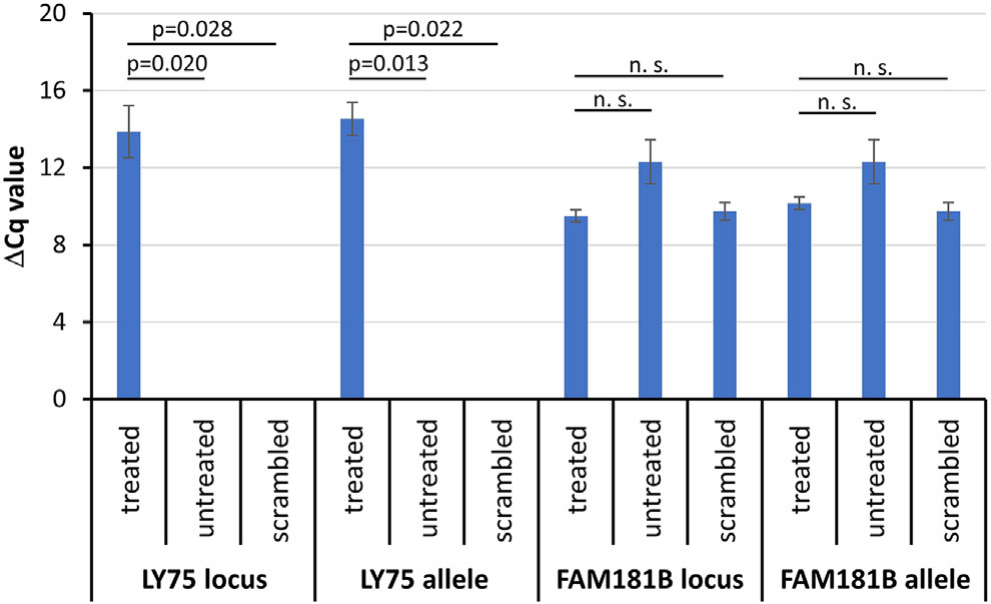
Expression levels of the LY75 and FAM181B 6 days after locus or allele-specific DNA demethylation Values provided are ΔCq values normalized with the housekeeping gene SDHA. The negative controls NRT (sample subjected to cDNA conversion without the enzyme reverse transcriptase) and NTC (no template control) showed no detectable signal for the target regions and SDHA. The Cq values for SDHA were in the range of cycle 22–26 in all experiments. The experiments were conducted in three biological replicates (except for FAM181B-locus where only two replicates were obtained) and in each case, the Cq-value of sample was based on three technical replicates of the qPCR. The bars and errors report the average and standard deviation of the biological replicates. *p* values were determined by two-sided t test assuming equal variance. The untreated and scrambled sgRNA-EpiEditor treated sample collected on day 6 showed no detectable expression signal for the target genes. In this case, 40 cycles were used as limit of detection. n. s., not significant (*p* value >0.05).

Following the efficient demethylation of the target regions that were all selected to cover promoter regions of genes, we aimed to analyze the impact of the methylation changes on the expression of the respective genes. For this, cDNA of the samples were used for reverse transcription—quantitative PCR (RT-qPCR) based expression analysis. The ΔCq values were calculated by normalizing the samples with the house keeping gene succinate dehydrogenase complex flavoprotein subunit A (SDHA). Unfortunately, it was not possible to establish a reliable RT-qPCR assay for UPK3A despite several attempts, because of the appearance of by-products in the PCR. In the case of the LY75 gene, expression was undetectable in the untreated and scrambled-sgRNA-EpiEditor treated samples, which is in accordance with their high DNA methylation levels. However, after treating the cells with the locus- or allele-specific EpiEditors, expression signals were detected indicating an upregulation of gene expression (Figure 4). In case of FAM181B, the expression levels of locus-specific and allele-specific treated samples were increased, but the effect was not significant and comparable to changes observed in the scrambled-sgRNA treated samples (Figure 4) indicating that no specific expression changes were triggered by the ASD. The lack of effects of the efficient locus-specific and ASD in FAM181B on gene expression could be due to the fact that the target region is more than 1000 bp downstream of the TSS, which may already be too far away from the promoter for DNA demethylation to have an effect on gene expression. Alternatively, DNA demethylation alone might be insufficient to stimulate FAM181B expression. However, we conclude that at least in the case of LY75, which is not expressed in untreated cells or cells treated with scrambled sgRNA, our data demonstrate a strong upregulation of gene expression after locus and ASD.

### Effect of demethylation on the allelic ratio of target gene expression

To further analyze the allele-specific expression of the target genes, we used an amplicon-based transcript sequencing to monitor the allelic ratios of gene expression. As the qPCR analysis already indicated a lack of specific expression changes for FAM181B, the allelic expression analysis experiments were only conducted for LY75 and UPK3A. For both genes, primers were designed to amplify an exonic region that contains an additional SNP. Then, the genomic sgRNA/dCas9 complex target region and the exon with the additional SNP were amplified and sequenced. The sequences were segregated based on their allelic read ratios allowing to pair the SNPs at the sgRNA binding position and in the exons based on the ratio of their allelic reads (Figures S8 and S9). These analyses showed that the allele frequency of the target alleles is 0.32 for LY75 and 0.73 for UPK3A at the genomic level, which is compatible with the copy numbers of the LY75 and UPK3A genes provided in the HEK293 database (3.15 and 4.28, respectively^29^).

**Figure 5:**
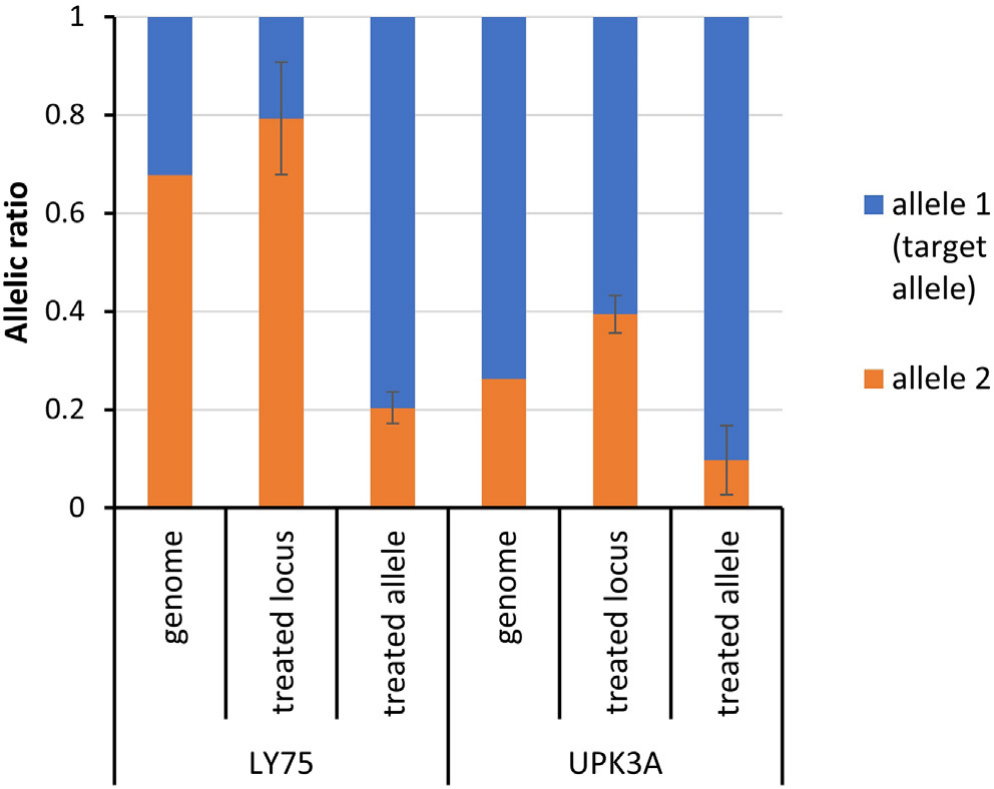
Genomic allelic ratio and ratio of allelic gene expression after locus and allele-specific DNA demethylation The expression analyses were conducted in biological triplicates, the error bars indicate the standard deviation. The samples subjected to locus and allele-specific demethylation treatment are labeled as “treated locus” and “treated allele”, respectively. The allele targeted in the allele-specific demethylation is referred as allele 1 in this graph. *p* values for the comparison of locus- and allele-specific treatment are 1.0x10^−3^ (LY75) and 0.036 (UPK3A), all based on two-sided t test assuming equal variance.

Next, we aimed to investigate the allelic ratios of transcript expression. As the expression of both target genes is very low in HEK293 cells (see last chapter and also GSE144903 ^34^), we did not aim to determine allelic expression levels of untreated cells, but compared the allelic expression ratio of the target genes after locus- and allele-specific DNA demethylation. As shown in Figure 5, the expression level of the LY75 target allele after locus-specific demethylation was 0.21, little less than the expected fraction of 0.32 based on the genome allele frequency. However, after allele-specific DNA methylation, the fraction of this allele in the expressed RNAs raised to 0.80, indicating a massive about 4-fold increase in allele-specific expression. In the case of UPK3A the target allele is overrepresented at the genomic level (allele frequency 0.73), which reduces the dynamic range of the detection. After locus-specific DNA demethylation the allelic expression ratio of this allele was 0.61, but it increased to 0.90 after ASD. The strong shift in allelic expression ratio is clearly visible at the expression of the non-target allele, which has a fraction of 0.39 after locus-specific demethylation, but drops 4-fold below 0.1 after the allele-specific epigenome editing. We conclude that in both cases, LY75 and UPK3A, strong (around 4-fold) changes in allelic expression ratios are triggered by ASD.

## Discussion

In this work, we showed the efficient, durable, ASD of target loci containing an SNP in the dCas9 PAM site. Strikingly, DNA demethylation at the targeted allele reached up to 90%, it was up to 15-fold more efficient than at the off-target allele and demethylation was almost stable over 15 days of the experiment. In epigenome editing, undesired off-target activity can result from binding of the sgRNA/dCas9 complex to the off-target allele, or untargeted activity of the EpiEditor enzymatic part. In our study, the off-target binding of the sgRNA/dCas9 was tested using predicted off-target sites of the different used sgRNA/dCas9 complexes with low sequence variance from the target regions (LRRK1, CTPB1-AS2, RP_11, SDF4, TUBB2B, and PCDH19). Activity by the freely diffusing EpiEditor was tested using scrambled sgRNA that does not have a target site in the human genome and by analysis of unrelated loci (SLC6A3 and MEST). Our data indicate that the off-target locus and off-target allele DNA demethylation was mainly caused by an untargeted activity of the TET1CD complex, similarly as observed in a previous work in allele-specific DNA methylation (ASM).^22^ Of note, the untargeted activity at each specific genomic locus depends on the accessibility of the locus, which explains the differences in off-target effects of the different loci that were investigated. Based on these findings, one promising approach to further reduce the off-target effects in locus and allele-specific epigenome editing could be a better control of the expression or activity of the epigenome editors, because too high expression and activity will inherently compromise the specificity of epigenome editing.

An interesting difference in the results of ASM^22^ and ASD (this work) experiments was in the kinetics of the effects. Although both studies were conducted with comparable epigenome editing platforms and in the same cell line, the strongest ASM effects were observed already 3 days after transfection, while ASD was highest 6 days after transfection. This delay in ASD could be related to the fact that DNA demethylation by TET1 requires multiple steps of the 5-methylcytosine oxidation followed by the final removal of the modified base, whereas DNA methylation is a direct one-step process. We observed high stability of the targeted DNA demethylation for up to 15 days although we showed in our previous studies that the expression of the EpiEditors was lost after around 9 days.^27^ Hence, the epigenome reprogramming was stable for several cell divisions even in the absence of the EpiEditors.

As a vision, potential clinical applications of ASEE could be in the correction of abnormal imprinting patterns. Genomic imprinting is found in about 150 mammalian genes many of which are involved in growth control and development.^35^^,^^36^ Imprinted genes are expressed only from the maternal or the paternal allele, which is triggered by an allele-specific DNA methylation at so called imprinting control regions (ICR). Aberrant DNA methylation at ICRs can lead to diseases such as cancer, imprinting disorders, autoimmune, metabolic, or neurological diseases.^37^^,^^38^ One example of an imprinting disease caused by abnormal hypermethylation of an ICR in the maternal genome is Beckwith-Wiedemann syndrome (BWS) (OMIM #130650) that is associated with increased birth weight and somatic overgrowth and predisposes for embryonal malignancies.^39^ About 5–10% of the diagnosed BWS cases, are purely epigenetic and contain DNA hypermethylation at the maternal ICR^40^ which could be directly corrected by ASD. Along the same lines, Kagami-Ogata syndrome (KOS) (OMIM #608149) is an imprinting disorder that in some cases is caused by gain of methylation in the MEG3 ICR.^41^ Hence, it can be addressed by targeted allele-specific demethylation in the same way, which in any case should happen as early as possible. Another interesting scenario is in the context of X chromosome inactivation in females.^42^ If the active X chromosome contains a disease mutation in one gene, reactivation of the allele located on the inactive X chromosome could be beneficial, similarly as recently shown for the activation of the MECP2 gene in Rett syndrome neurons.^43^ Finally, in basic research the novel technologies of allele-specific DNA methylation^22^ and demethylation (this paper) provide valuable additions to the experimental toolbox, potentially allowing researchers to directly investigate biological effects of different alleles in a given cellular background.

### Limitations of the study

The current experiments were conducted in HEK293 cells. It has not been investigated if results can be generalized to other cell lines. While the epigenome editing complexes used in our study are not dependent on cellular factors, the further processing of the oxidized 5mC residues finally leading to DNA demethylation and the recruitment of DNMT activity to the target sites potentially reverting the demethylation could vary in different cell types. Moreover, the expression levels of the epigenome editors may vary with the cell line, leading to different efficiencies of on-target editing and changes in the editing specificity. Of note, ASD depends on the presence of an SNP as part of the PAM or sgRNA binding site in an expression controlling genetic element. This needs to be identified and validated for each potential application. On average, two human genomes differ from each other at every 1000 bases^44^ and SNPs occur on a population level with a frequency of one per 300 bps. Statistically, some SNPs are in heterozygous states in individuals and then allow for the discrimination of alleles. The effects of ASD on allelic transcription, depend on the cell-type specific availability of transcription factors and potential effects of the chromatin environment. ASD could only be considered for treatment of imprinting disorders caused by the abnormal hypermethylation of an imprinting locus in both alleles.

## Resource availability

### Lead contact

Further information and requests for resources should be directed to and will be fulfilled by the lead contact, Albert Jeltsch (albert.jeltsch@ibtb.uni-stuttgart.de).

### Materials availability

This study did not generate new unique reagents.

### Data and code availability

All data reported in this paper will be shared by the lead contact upon request. All original sequencing data have been uploaded to the DARUS repository (https://doi.org/10.18419/darus-4230). Any additional information needed to reanalyze the data reported in this paper is available from the lead contact upon request.

## Acknowledgments

This work has been supported by the 10.13039/100008316Baden-Württemberg Stiftung gGmbH (AllEpi, ID09 to A.J.). The funder had no role in the design of the study and collection, analysis, and interpretation of data and in writing the manuscript which should be declared.

## Author contributions

P.B. and A.J. devised the study. N.R. conducted all experiments with help from K.B. N.R., P.B., and A.J. were involved in data analysis. P.B. and A.J. supervised the work. N.R., P.B., and A.J. wrote the draft article. N.R. prepared the figures. A.J. acquired funding. All authors were involved in preparing the final manuscript.

## Declaration of interests

The authors declare that they have no competing interests.

## STAR★Methods

### Key resources table

**Table:** 

REAGENT or RESOURCE	SOURCE	IDENTIFIER
**Bacterial and virus strains**		

XL1-Blue recA1 endA1 gyrA96 thi-1 hsdR17 supE44 relA1 lac [F proAB lacIqZΔM15 Tn10 (Tetr)]	Agilent	200249

**Chemicals, peptides, and recombinant proteins**		

Dulbecco’s Modified Eagle’s Medium (DMEM)	Sigma	D5671
Fetal Bovine Serum	Sigma	F7524
L- Glutamine	Sigma	G7513
PenStrep	Sigma	P0781
Trypsin	Sigma	T3924
PBS	Sigma	D802
FuGENE	Promega	E5911
EcoRV	NEB	R3195

**Critical commercial assays**		

EZ DNA Methylation-Lightning Kit	Zymo	D5030-E
SPRIselect Bead-Based Reagent	Beckman Coulter	B23317
RNeasy extraction kit	Qiagen	74034
TURBO DNA-free™ Kit	Ambion	AM1907
High-Capacity cDNA Reverse Transcription Kit	Applied Biosystems	4368814
ORA™ SEE qPCR Probe Mix	highQu	QPP0401

**Deposited data**		

Primary Sequencing data	This paper	https://doi.org/10.18419/darus-4230
MBD-seq	Hofacker et al.^20^	https://www.ncbi.nlm.nih.gov/geo/query/acc.cgi?acc=GSE144331
SNP	Lin et al.^29^	http://hek293genome.org/v2/data.php

**Experimental models: Cell lines**		

HEK293	DSMZ (Braunschweig, Germany)	RRID: CVCL_0045

**Oligonucleotides**		

Oligonucleotides used for sgRNA cloning	This Paper	See Table S2
Primers used for multi-sgRNA cloning	This Paper	See Table S3
Target specific primers used for amplifying bisulfite treated gDNA samples	This Paper	See Table S4
Primers with Illumina adapters used in the second PCR reaction for analysis of bisulfite treated gDNA	This Paper	See Table S5
qPCR primers used for expression analysis	This Paper	See Table S6
Primers used for amplification of transcripts	This Paper	See Table S7
Primers used for amplification of genomic regions	This Paper	See Table S8

**Recombinant DNA**		

pCAG-dCas9-5xPlat2AflD	Morita et al.^24^	Addgene plasmid #82560
scFv-GCN4-DNMT3a-DNMT3l	Hofacker et al.^20^	Addgene plasmid #154140
FH-TET1-pEF	Tahiliani et al.^6^	Addgene plasmid #49792
pCAG-scFvGCN4sfGFPTET1CD	Morita et al.^24^	Addgene plasmid #82561
sgRNA_expression_vector	Albrecht et al.^27^	Addgene plasmid #210212
pMulti-sgRNA-LacZ-DsRed vector	Shao et al.^45^	Addgene plasmid #99914

**Software and algorithms**		

CFX Maestro Software 2.3 for Windows PC	Bio-Rad	#12013758
Trim Galore	Babraham Bioinformatics	https://www.bioinformatics.babraham.ac.uk/projects/trim_galore/
liftOver	UCSC	https://genome.ucsc.edu/cgi-bin/hgLiftOver
fastq_filter	Blankenberg et al.^46^	https://github.com/galaxyproject/sequence_utils
fastq_to_tabular	Blankenberg et al.^46^	https://github.com/galaxyproject/sequence_utils
PEAR	Zhang et al.^47^	https://github.com/tseemann/PEAR
bwameth	Pedersen et al.^48^	https://github.com/brentp/bwa-meth
MethylDackel	MIT license https://github.com/dpryan79/MethylDackel.git	https://github.com/dpryan79/MethylDackel.git
CCTOP	Stemmer et al.^32^	https://cctop.cos.uni-heidelberg.de/
Cas-offinder	Bae et al.^33^	http://www.rgenome.net/cas-offinder/

### Experimental model and study participant details

#### Cell culture

Human embryonic kidney (HEK293; female; RRID: CVCL_0045) cells were obtained from DSMZ (Braunschweig, Germany). The cells were cultivated in DMEM culture medium (DMEM supplemented with 10% Fetal Bovine Serum (FBS), 1% penicillin/streptomycin, 4 mM L-glutamine) in a humidified incubator (BINDER) at 37°C and 5% CO_2_. To maintain a confluency of 70-90%, cells were split at a 1:7 to 1:10 ratio every 2 to 3 days. For this, the cells were washed with PBS not containing CaCl_2_ and MgCl_2_, followed by addition of trypsin-EDTA solution (Sigma) and incubation at 37°C until cells detached. Afterward, cells were resuspended using culture medium and split in the desired ratio. For storage, cells were harvested at 300 g for 5 min, resuspended in freezing medium (90% FBS, 10% DMSO) and gradually frozen to -80°C. Cells were routinely checked for mycoplasma contamination by PCR.^49^

### Method details

#### Plasmid generation

The dCas9-5X SunTag-scFv-TET1CD-IRES-GFP vector was generated by fusing dCas9-5X SunTag region amplified from the Addgene Plasmid #82560 and the insert scFv-TET1CD-IRES-GFP. The dCas9-5X SunTag region was cloned under SV40 promoter and the insert scFv-TET1CD-IRES-GFP was cloned under the SFFV promoter. The insert scFv, TET1CD and IRES-GFP were amplified from the Addgene plasmids #154140, #49792 and #82561, respectively.^27^ The sgRNA vectors for each target were generated by inserting the target sequences into the Addgene Plasmid #210212.^50^ The sgRNA expression vector generated to contain the sgRNA sequence flanked by the U6 promoter and the sgRNA scaffold. The oligonucleotides used for sgRNA cloning are provided in the Table S2. The regions comprising U6 promoter, sgRNA sequence and sgRNA scaffold were amplified from each plasmid with primers designed to provide suitable overhangs (Table S3). The amplified sequences were inserted into the pMulti-sgRNA-LacZ-DsRed vector (a gift from Yujie Sun, Addgene plasmid # 99914 by Golden Gate Assembly to generate a multi-sgRNA expression vector. The multi-sgRNA vector co-expresses the fluorophore DsRed. A scrambled sgRNA (GAACAGTCGCGTTTGCGACT) was used as a negative control which does not have a binding site in the human genome.^20^^,^^31^

#### Cell transfection

Transient transfection of the plasmids was performed with transfection reagent FuGENE® HD as per the manufacturer’s instructions. About 1.4 million cells were seeded in 100 mm dish a day prior to the transient transfection. On the day of transfection, the confluent cells were co-transfected with 9000 ng of the dCas9-5X SunTag-scFv-TET1CD and 500 ng of the multi-sgRNA expression plasmids. After 24 hours, the growth medium was replaced with the fresh medium. The cells were harvested and filtered with 30 μm (Cat no: 130-041-407, Miltenyi Biotec). The filtered cells were sorted by SH800S Cell Sorter (Sony Biotechnology) to enrich the cells positive for both plasmids. A fraction of the sorted cells was centrifuged at 100 rpm for 5 min at RT and stored at -80°C for further use and the other fraction of cells were cultured until day 15. The cells were sub-cultured every 3^rd^ day and the samples were collected on the days 3, 6, 9 and 15. The cells were collected by centrifugation (100 rpm for 5 min at RT) and stored at -80°C for downstream experiments.

#### Bisulfite library preparation

Genomic DNA was isolated with QIAmp DNA Mini Kit (Qiagen). 500 ng of the genomic DNA was subjected to an overnight enzymatic fragmentation by EcoRV (NEB Catalog #R0195L) which does not cleave within any of the genomic regions of interest. The digested genomic DNA was bisulfite converted with the Zymo EZ DNA Methylation-Lightning Kit (D5030-E). The bisulfite treated samples were amplified with primers specific for the desired region with barcodes and adapters (Table S4). The regions amplified using the primers are provided in Figure S1. The product of the PCR was used as template for the following PCR reaction. The primers used for PCR2 add the Illumina adapter sequences required for NGS sequencing (Table S5). The presence of barcode and unique indices enabled multiplexed NGS sequencing. The products of the second PCR were pooled in equimolar amounts and purified with SPRIselect beads (Beckman Coulter). The purified ready to use pools of libraries were sequenced on a NovaSeq 6000 system using a PE250 run (Novogene).

#### NGS data analysis

The NGS data received as FASTQ files, were processed in the local Galaxy Server.^46^^,^^51^^,^^52^ Trim Galore! was used for adapter and quality trimming. (https://www.bioinformatics.babraham.ac.uk/projects/trim_galore/). PEAR^47^ was used for merging paired reads. The de-multiplexing was done by the selection of the samples with specific combinations of barcodes and Illumina adapters. Reads corresponding to different alleles of the same target gene were selected by filtering the reads with the SNP nucleotide sequence tag. Two files of reads corresponding to alleles were generated and their DNA methylation level was analyzed independently. First, reads were mapped against the reference genome hg19 using bwameth^48^ and then DNA methylation of individual CpG sites was computed using MethylDackel (https://github.com/dpryan79/MethylDackel). For this the standard hg19 reference genome was used corresponding to allele 2 of LY75 and FAM181B and allele 1 of UPK3A. Final visualization and statistics were prepared using Microsoft Excel.

#### Analysis of DNA demethylation

For quantitative analysis of DNA demethylation efficiency, the CpG sites with at least 50% of the highest demethylation effect were selected for each target and their methylation levels averaged. The same set of CpG sites was used for the analysis of all samples of the corresponding experiment (untreated, scrambled, and treated). DNA demethylation efficiencies (D, in %) are given relative to the methylation levels in the control cells.

$$D=\left(M_{\text{control}}-M_{\text{treated}}\right)/M_{\text{control}}$$

with: M_control_: DNA methylation levels of the untreated samples (in %), M_treated_: DNA methylation levels of the treated samples (in %).

#### RNA isolation

RNA was isolated from the samples using Qiagen RNeasy extraction kit (Cat. No. 74034). Prior to cDNA conversion, RNA samples were subjected to DNase treatment to remove any residual genomic DNA using TURBO DNA-free™ Kit (Ambion #AM1907). 500 ng of the purified RNA was used for cDNA synthesis with Applied Biosystems- High-Capacity cDNA Reverse Transcription Kit (Cat No 4368814). cDNA synthesis without the reverse transcriptase enzyme was conducted as a negative control for all samples and referred as NRT. In addition, control samples were prepared not including template RNA and referred as NTC.

#### Quantitative PCR for expression analysis

The qPCR reaction was conducted with 7.5 μL 2× ORA™ See qPCR Probe Mix, 0.4 μL forward primer (10 μM), 0.4 μL reverse primer (10 μM), 2 μL of cDNA (undiluted cDNA) and ddH_2_O was used to make up the volume to 15 μL. The primer sequences are given in Table S6. The conditions used for qPCR are as follows: 95°C for 3 min, 40 cycles of 95°C for 15 s, followed by T_A_ (annealing temperature) for 30 s, 95°C for 10 s, and lastly a 65–95°C ramp (0.5°C steps every 5 s) to generate the melting curve. The transcript levels of the samples were analyzed by normalizing the Cq values to the reference gene Succinate Dehydrogenase Complex Flavoprotein Subunit A (SDHA) using the −ΔCq calculation. The experiments were conducted in biological triplicates and each sample Cq value was based on three technical replicates of the qPCR.

#### Transcript sequencing

To distinguish the transcripts in the sequencing, primers were designed to amplify an exonic region of the gene that contains a heterozygous SNP (Table S7). The cDNA samples were amplified with the target region specific primers using Hot start Taq Polymerase (95 C for 15min, 35 cycles of 94 C—30s, T_A_—30s and 72°C for 60 s, and 72 C for 10 min). The products of the PCR were used as a template for the subsequent PCR2. The primers used for the PCR2 incorporate adapters required for Illumina sequencing to the product of PCR1. Q5 polymerase was used for amplification (98°C for 30 s, 15 cycles of 98°C for 10s and 72 for 40 s, and 72°C for 2 min).

#### Genomic region sequencing

To establish the connection between the SNP in the sgRNA targeted region and an exon in the gene with an additional SNP that were used in the transcript sequencing, the genomic regions of the respective sites were amplified and sequenced. Primers used to amplify the respective regions are provided in Table S8. 10 ng of the genomic DNA was used for two-step library generation as described for transcript sequencing. The genomic DNA were amplified with the target region specific primers using Hot start Taq Polymerase (95°C for 15min, 35 cycles of 94°C—30s, T_A_—30s and 72°C for 60 s, and 72°C for 10 min). The products of the PCR were used as a template for the subsequent PCR2. The primers used for the PCR2 (Table S5) incorporate adapters required for Illumina sequencing to the product of PCR1. Q5 polymerase was used for amplification (98°C for 30 s, 15 cycles of 98°C for 10s and 72 for 40 s, and 72°C for 2 min). Sequence reads were split based on the presence of the SNP and counted to determine the genomic allele frequencies.

### Quantification and statistical analysis

The number of experimental repeats is indicated for each experiment. P-values were derived by two-sided T-test with equal variance using MS Excel.
