# Supplementary material for: Allele-specific DNA demethylation editing leads to stable upregulation of allele-specific gene expression
Source: iScience. 2024 Sep 23;27(10):111007. doi: 10.1016/j.isci.2024.111007 (PMC11490731; doi:10.1016/j.isci.2024.111007)
Supplement: Document S1. Figures S1–S9 and Tables S1–S8 [file mmc1.pdf]

iScience, Volume *27*

Supplemental information

Allele-specific DNA demethylation editing leads to stable upregulation of allele-specific gene expression

Nivethika Rajaram, Katharina Benzler, Pavel Bashtrykov, and Albert Jeltsch

Supplementary Figures and Tables

Figure S1: Schematic images representing the design of locus-specific and allele-specific sgRNAs, related to Figure 1. The positions of the locus-specific sgRNA and the allele-specific sgRNA binding site are shown. The PAM (NGG) is indicated in green text. The SNP is highlighted in red color. The locus-specific sgRNAs (brown bar) do not discriminate between both the alleles and possess a matching binding region in both alleles. In case of allele-specific targeting, the targeted allele has a potential PAM site enabling the binding of the sgRNA/dCas9 complex, whereas the presence of SNP in the untargeted allele disrupts the potential PAM, thereby disfavoring the binding of the sgRNA/dCas9 complex.

LY75 sgRNA design

sgRNA locus-specific TTATATAAGAGAGGCGACGA

sgRNA allele-specific AGAGAGGCGACGATGGAGCA

Allele 1

```
                        AGAGAGGCGACGATGGAGCA
                 TTATATAAGAGAGGCGACGA       ↓
5'CATACTCCAGCAACATTATATAAGAGAGGCGACGATGGAGCAGGGCACCCGGCCAAAAAGCCTC 3'
  ||||||||||||||||||||||||||||||||||||||||||||||||||||||||||||||||
3'GTATGAGGTCGTTGTAATATATTCTCTCCGCTGCTACCTCGTCCCGTGGGCCGGTTTTTCGGAG 5'
                                     PAM    PAM
```

Allele 2

```
                 TTATATAAGAGAGGCGACGA       ↓
5'CATACTCCAGCAACATTATATAAGAGAGGCGACGATGGAGCAGCGCACCCGGCCAAAAAGCCTC 3'
  ||||||||||||||||||||||||||||||||||||||||||||||||||||||||||||||||
3'GTATGAGGTCGTTGTAATATATTCTCTCCGCTGCTACCTCGTCGCGTGGGCCGGTTTTTCGGAG 5'
                                     PAM
```

FAM181B sgRNA design

sgRNA locus-specific CCCGCCGGGAGAATCCGCAG

sgRNA allele-specific GCGAAAGAGGCCAAATGGCC

Allele 1

```
        GCGAAAGAGGCCAAATGGCC ↓          CCCGCCGGGAGAATCCGCAG
5'AAGGGGGCGAAAGAGGCCAAATGGCCCGGCCCGTCCTCCCCGCCGGGAGAATCCGCAGCGGCGGG 3'
  |||||||||||||||||||||||||||||||||||||||||||||||||||||||||||||||||
3'TTCCCCCGCTTTCTCCGGTTTACCGGGCCGGGCAGGAGGGGCGGCCCTCTTAGGCGTCGCCGCCC 5'
                            PAM                               PAM
```

Allele 2

```
                             ↓          CCCGCCGGGAGAATCCGCAG
5'AAGGGGGCGAAAGAGGCCAAATGGCCCCGCCCGTCCTCCCCGCCGGGAGAATCCGCAGCGGCGGG 3'
  |||||||||||||||||||||||||||||||||||||||||||||||||||||||||||||||||
3'TTCCCCCGCTTTCTCCGGTTTACCGGGGCGGGCAGGAGGGGCGGCCCTCTTAGGCGTCGCCGCCC 5'
                                                              PAM
```

UPK3A sgRNA design

sgRNA locus-specific CCCGGTGGGATTGCGCCCTG

sgRNA allele-specific GTAGGCGCTTTGATGCGGGC

Allele 1

```
      GTAGGCGCTTTGATGCGGGC ↓            CCCGGTGGGATTGCGCCCTG
5'CACAGTAGGCGCTTTGATGCGGGCTGGAGAAGGTCCTGCCCGGTGGGATTGCGCCCTGGGGCACC 3'
  |||||||||||||||||||||||||||||||||||||||||||||||||||||||||||||||||
3'GTGTCATCCGCGAAACTACGCCCGACCTCTTCCAGGACGGGCCACCCTAACGCGGGACCCCGTGG 5'
                          PAM                                 PAM
```

Allele 2

```
                           ↓            CCCGGTGGGATTGCGCCCTG
5'CACAGTAGGCGCTTTGATGCGGGCTCGAGAAGGTCCTGCCCGGTGGGATTGCGCCCTGGGGCACC 3'
  |||||||||||||||||||||||||||||||||||||||||||||||||||||||||||||||||
3'GTGTCATCCGCGAAACTACGCCCGAGCTCTTCCAGGACGGGCCACCCTAACGCGGGACCCCGTGG 5'
                                                              PAM
```

Figure S2: Compilation of the regions analyzed for DNA methylation by amplicon-based bisulfite sequencing, related to Figure 1. CpG sites are shaded in yellow. The numbering of the CpG sites and their position in the amplicon is shown in the tables below.

LY75

```
TGTGGCCAAGGTGACCAAGGGCCGAAGGAAAAGCGAGAACGGGAGGGACGGGACGCAAGA     60
GGGCAGATGGGGAACCCCATACTCCAGCAACATTATATAAGAGAGGCGACGATGGAGCAG    120
CGCACCCGGCCAAAAAAGCCTCCGTGCGCCTACTCTACGGTGCACCGCGTCCCCTCTGCA    180
CCAGAAGGGCCCTGTCCTCCCACATCCACCGCGCCCTCCTCCGGGCCCCCGAGGGCACTG    240
GGGCGCTTCCTCTGCCAGACCTCCCCTGCGACTCACTCTTCCGGCTCCAGAGCCCCCCCG    300
CCCCAACAGCAAAGCAGCCGTGACCTGCCCCAGGGGCGCAGCCCTGCCCCAGGCTGGAAG    360
GCAG
```

CpG site	1	2	3	4	5	6	7	8	9	10	11	12	13	14	15	16	17	18	19	20	21	22	23	24
Position	23	34	40	49	54	107	110	121	127	143	147	158	166	168	210	212	222	230	244	269	282	299	319	337

FAM181B

```
GATCTCCAAAGGCTGGAATAGGCGGTGCGGCTGTAGCCCGCGCTGTAATCGTAGGACACC     60
TGATGGGGCGGCGGCGGCGGGGGCAGGGCGCAGTCTGGAAAGAAGGGGGCGAAAGAGGCC    120
AAATGGCCCCGCCCGTCCTCCCCGCCGGGAGAATCCGCAGCGGCGGGGTACAGGGGGCTC    180
AAGGGCTCGTTCAAGGTCAAGCCGCCGCGGGGGGCAGTCAGGGGGCTCTTTTTGGTCGGG    240
GAGCAGCCCGGGACGCTCCAGGGCTCGGGGTACAGTAGGTTTCCCACCACTGCC
```

CpG site	1	2	3	4	5	6	7	8	9	10	11	12	13	14	15	16	17	18	19	20	21	22	23	24	25	26
Position	23	28	39	41	50	69	72	75	78	89	110	130	134	143	146	156	161	164	188	203	206	208	237	249	254	266

UPK3A

```
TCCCATTGGTGAGAGACGCTGTGGAAGGGCTGCCCTGGCCTGGGCACACAGTAGGCGCTT     60
TGATGCGGGCTGGAGAAGGTCCTGCCCGGTGGGATTGCGCCCTGGGGCACCGCGCACGGG    120
TCGGGGAAGCCCGGGGCAGGGCCGTATGCAAATAGCGCGCGAGGAGGCCGCGGATTGGCC    180
AGCCCAGGCGGGGGCGGGGCGCGCCGAGGGCCCAGAGCTGGCAGGTGCCCT
```

CpG site	1	2	3	4	5	6	7	8	9	10	11	12	13	14	15	16	17	18	19	20	21	22
Position	17	56	66	72	87	98	111	113	117	122	132	143	156	158	160	169	171	189	195	200	202	205

Figure S3: Schematic representation of the epigenome editing workflow used here, related to STAR Methods. The plasmids encoding dCas9 fused to the 5X SunTag, TET1CD fused to scFv together with GFP and sgRNAs together with DsRed are co-transfected into HEK293 cells. Three days post transfection, the double-positive cells are enriched using FACS. The sorted cells are cultured until Day 15 with collection of cells on days 3, 6, 9 and 15. Genomic DNA is isolated and subjected to bisulfite conversion followed by library preparation, Illumina sequencing, and data analysis.

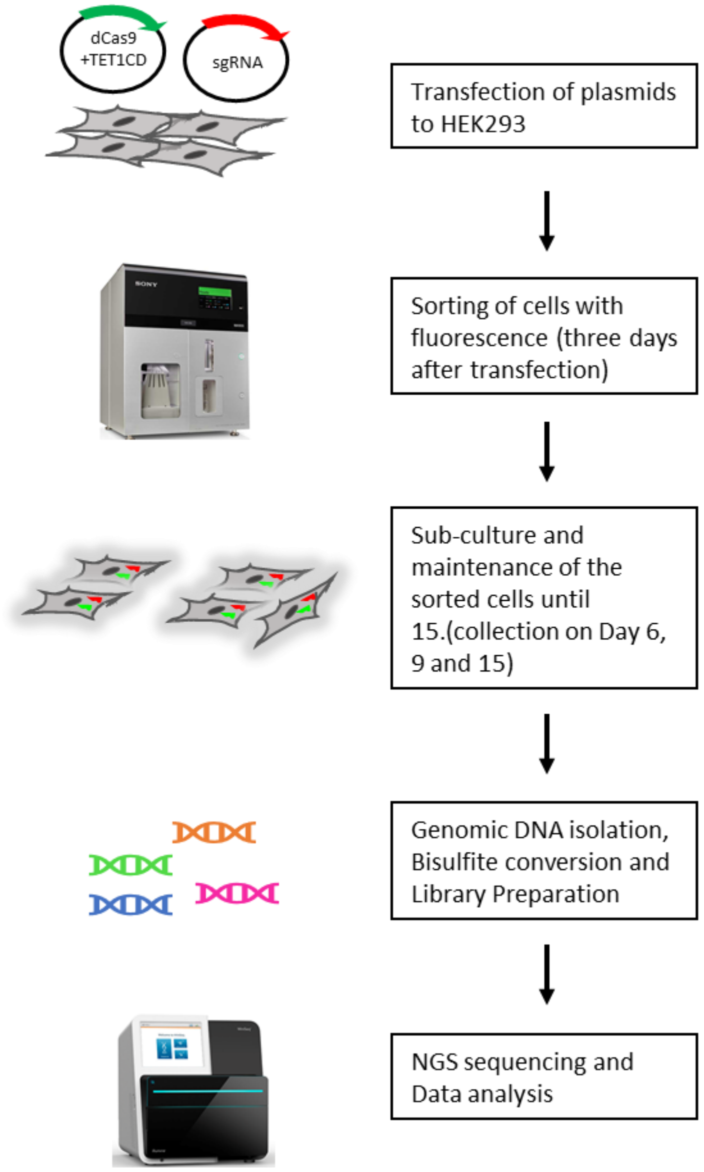

Figure S4: Methylation profile of the methylated off-target sites, related to Figure 2 and 3. (A) Methylation profiles of the off-target regions SLC6A3 and MEST after treatment with the locus-specific EpiEditors. (B) Quantitative analysis of the methylation at the analyzed region provided as a bar graph. Every CpG site included in the on-target analysis of the respective region was included. (C) Methylation profiles of the off-target regions SLC6A3 and MEST after treatment with the allele-specific EpiEditors. (D) Quantitative analysis of the methylation at the analyzed region provided as a bar graph. Every CpG site included in the on-target analysis of the respective region was included. These experiments were conducted in biological triplicates.

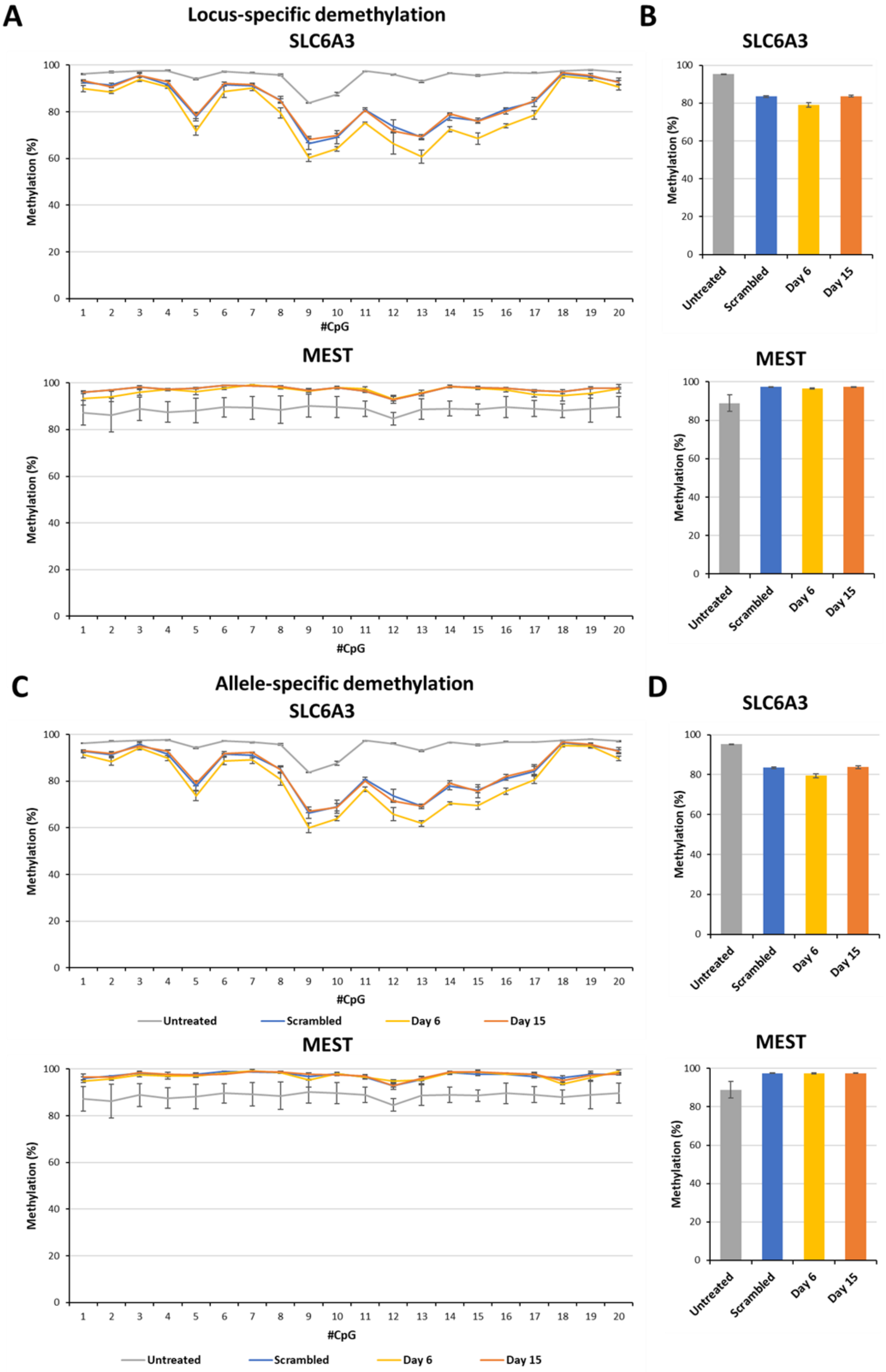

Figure S5: Methylation profiles of off-target sites potentially caused by off-target binding of the locus-specific demethylation sgRNAs, related to Figure 2. (A) The methylation profiles of samples treated with locus-specific sgRNA-EpiEditors were collected on day 6 and day 15 and labelled as Day 6 and Day 15, respectively. The sample treated with scrambled sgRNA-EpiEditors were collected on day 6 and labelled as 'Scrambled'. (B) Quantitative analysis of the methylation at the analyzed regions provided as bar graphs. Every CpG site included in the NGS analysis of the respective region were included. The experiments were conducted in biological triplicates.

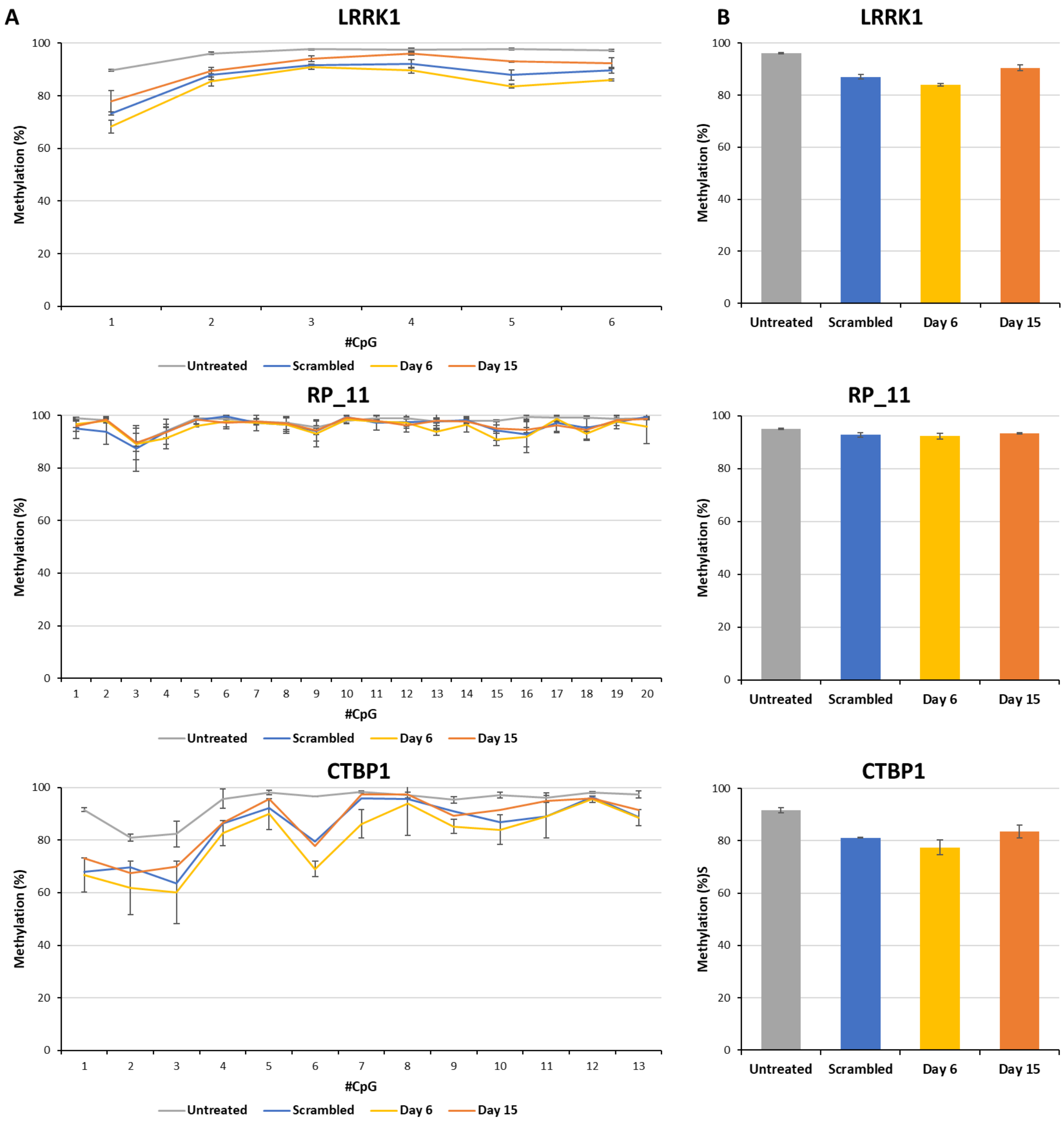

Figure S6: Methylation profiles of off-target sites potentially caused by off-target binding of the allele-specific demethylation sgRNAs, related to Figure 3. (A) The methylation profiles of samples treated with allele-specific sgRNA-EpiEditors were collected on day 6 and day 15 and labelled as Day 6 and Day 15, respectively. The sample treated with scrambled sgRNA-EpiEditors were collected on day 6 and labelled as 'Scrambled'. (B) Quantitative analysis of the methylation at the analyzed regions provided as bar graphs. Every CpG site included in the NGS analysis of the respective region were included. The experiments were conducted in biological triplicates.

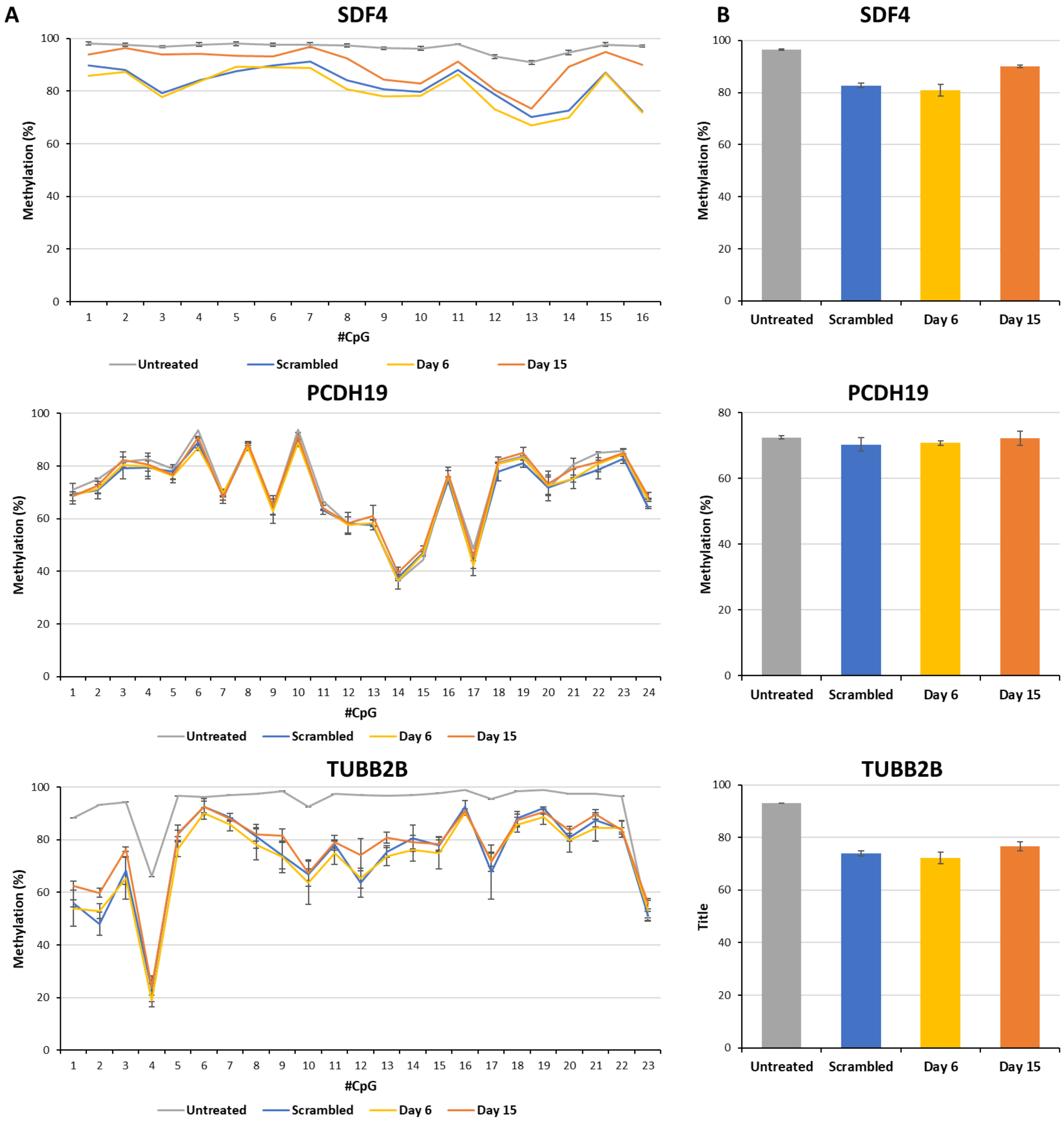

Figure S7: Allelic resolution of locus-specific demethylation, related to Figure 2 and 3. The bisulfite sequencing data of the samples subjected locus-specific demethylation (shown in Figure 2) at day 6 were analysed at allelic levels. The experiment was conducted in biological triplicates.

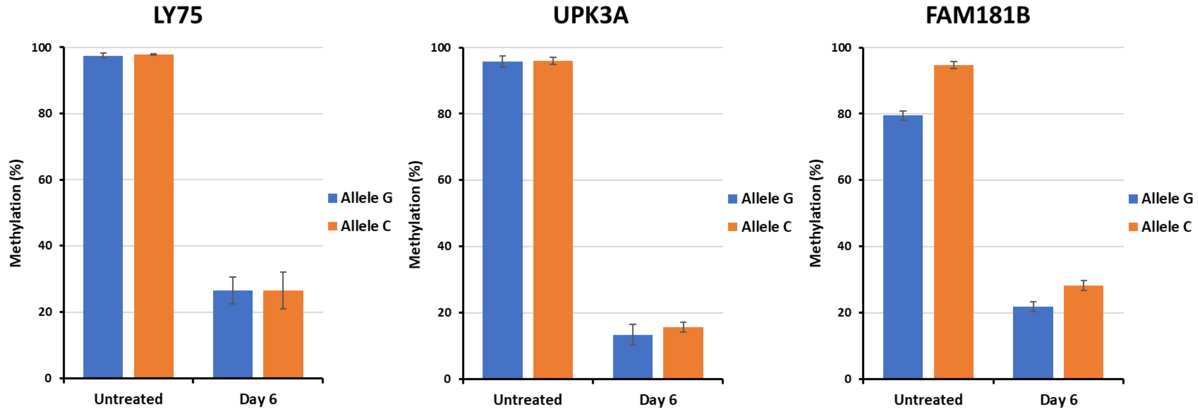

Figure S8: Scheme of allelic arrangement with SNPs at the LY75 locus, related to Figure 5. (A) The regions with SNPs in the sgRNA binding site (purple line) and part of exon 18 (yellow) were amplified as indicated with red arrows and sequenced. The ratio of the alleles in the respective sequencing is provided in the table below. (B) The chromosomal arrangement of the alleles with respective paired SNPs established based on the allelic ratios obtained from the table in panel A. In allele 1, the 'G' in the sgRNA binding region is paired to the 'A' in exon 18. In allele 2, the 'C' in the sgRNA binding position is paired to the 'G' in exon 18. (C) Scheme of locus-specific demethylation. The sgRNA targeting the region binds to allele 1 and 2 without discrimination leading to demethylation on both alleles. The transcription should be initiated on both alleles. For allelic expression analysis, exon 18 is amplified with a pair of primers as indicated with red arrows. (D) Scheme of allele-specific demethylation. The sgRNA targets allele 1 specifically and should initiate transcription of this allele.

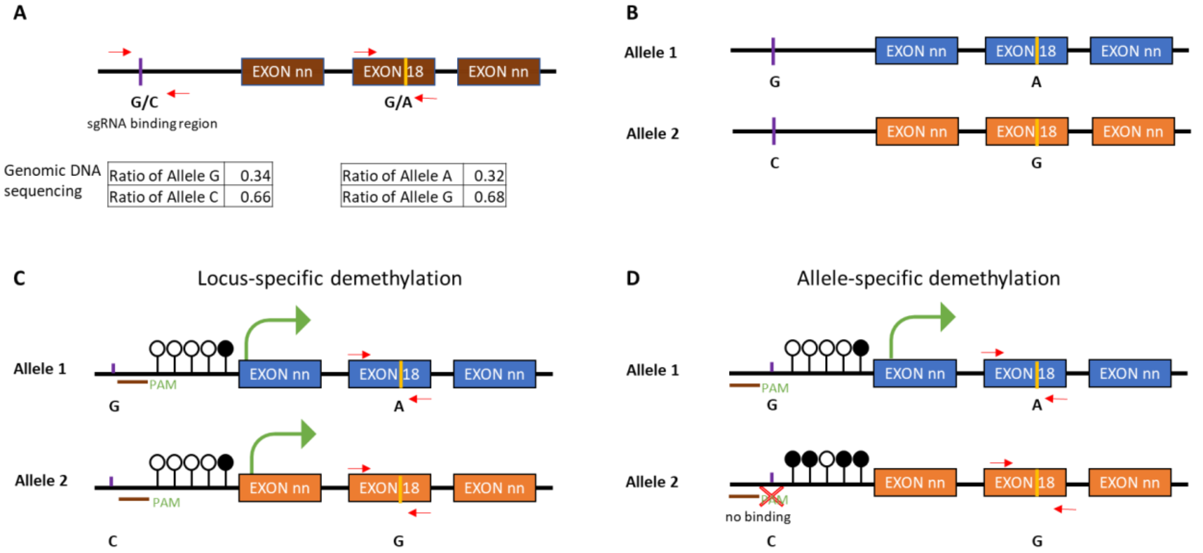

Figure S9: Scheme of allelic arrangement with SNPs at the UPK3A locus, related to Figure 5. (A) The regions with SNPs in the sgRNA binding site and part of exon 4 (highlighted with purple and yellow lines, respectively) were amplified as indicated with red arrows and sequenced. The ratio of the alleles in the respective sequencing is provided in the table below. (B) Chromosomal arrangement of the alleles with respective paired SNPs established based on the allelic ratios obtained from the table in panel A. In allele 1, the 'G' in the sgRNA binding region is paired to the 'G' in exon 4. In allele 2, the 'C' in the sgRNA binding position is paired to the 'A' in exon 4. (C) Scheme of locus-specific demethylation. The sgRNA targeting the region binds to allele 1 and 2 without discrimination leading to demethylation on both alleles. The transcription should be initiated on both alleles. For allelic expression analysis, exon 4 is amplified with a pair of primers as indicated with red arrows. (D) Scheme of allele-specific demethylation. The sgRNA targets allele 1 specifically and should initiate transcription of this allele.

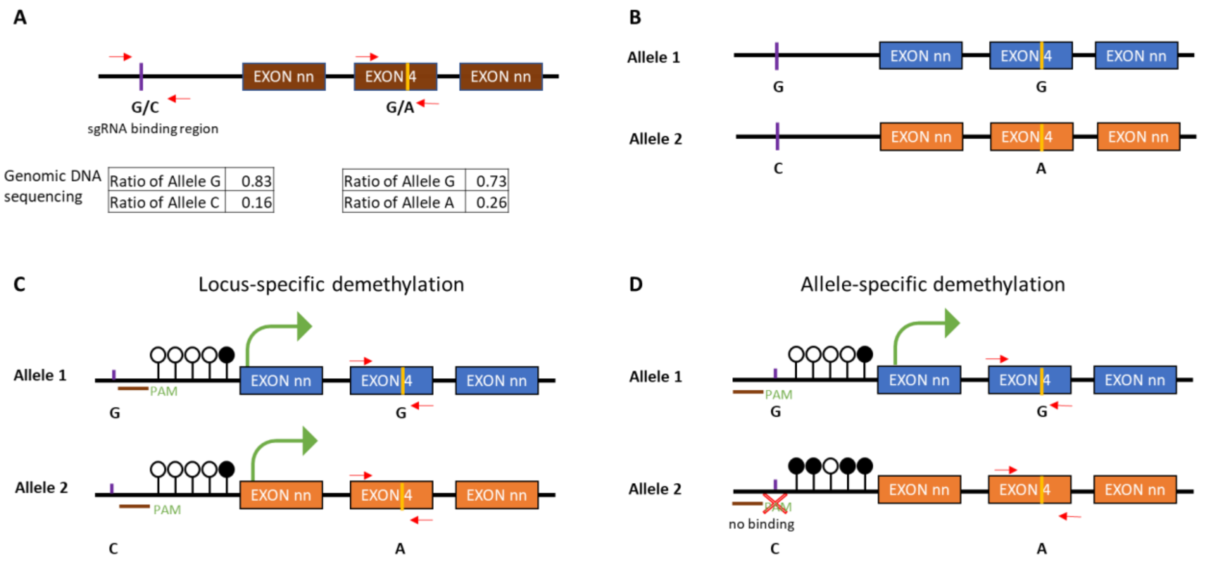

Table S1: List of analyzed potential sgRNA/dCas9 complex off-target binding sites, related to STAR Methods. The mismatches are highlighted in red. The genomic coordinates (hg19) of the potential binding site are provided together with the number of CpG sites in the analyzed off-target region.

**Target region**	**LY75 locus**	**UPK3A locus**	**FAM181B locus**	**LY75 allele**	**UPK3A allele**	**FAM181B allele**
Analyzed off-target region	LRRK1	RP_11	CTBP1-AS2	SDF4	PCDH19	TUBB2B
sgRNA sequence (20 nt + PAM)	TTATATAAGAGAGGCGACGATGG	GTAGGCGCTTTGATGCGGGCTGG	CCCGCCGGGAGAATCCGCAGCGG	AGAGAGGCGACGATGGAGCAGGG	CCCGGTGGGATTGCGCCCTGGGG	GCGAAAGAGGCCAAATGGCCCGG
Sequence of the potential binding region with mismatches (20 nt + PAM)	TTATAGTAGAGATGAGACGATGG	GCAGGAGCCTTGAGGCGGGCGGG	CCCCTCGGGAGAGTCTGCAGGGG	AGAGAGGCAGCGATGGAGGCGGG	CCCGCTTGGAATGCGCCCTCCGG	GCGAGAAAGGCCGAATGACCCGG
Genomic co-ordinates of the potential off-target binding region (hg 19, 20 nt + PAM)	chr15: 101541523-101541545	chr9: 97094730-97094752	chr4: 1243762-1243784	chr1: 1155672-1155694	chrX: 99664157-99664179	chr6: 3229438-3229460
# of CpG sites in the analyzed off-target region	5	7, 8, 9	7	2,3	11,12	13,14

Table S2: List of oligonucleotides used for sgRNA cloning, related to STAR Methods.

Gene	sgRNA Sequence	Description
LY75	accgAGAGAGGCGACGATGGAGCA	LY75 Allele-specific sgRNA sense oligonucleotide
LY75	aaacTGCTCCATCGTCGCCTCTCT	LY75 Allele-specific sgRNA antisense oligonucleotide
LY75	accgTTATATAAGAGAGGCGACGA	LY75 Locus-specific sgRNA sense oligonucleotide
LY75	aaacTCGTCGCCTCTCTTATATAA	LY75 Locus-specific sgRNA antisense oligonucleotide
FAM181B	accgGCGAAAGAGGCCAAATGGCC	FAM181B Allele-specific sgRNA sense oligonucleotide
FAM181B	aaacGGCCATTTGGCCTCTTTCGC	FAM181B Allele-specific sgRNA antisense oligonucleotide
FAM181B	accgCCCGCCGGGAGAATCCGCAG	FAM181B Locus-specific sgRNA sense oligonucleotide
FAM181B	aaacCTGCGGATTCTCCCGGCGGG	FAM181B Locus-specific sgRNA antisense oligonucleotide
UPK3A	accgGTAGGCGCTTTGATGCGGGC	UPK3A Allele-specific sgRNA sense oligonucleotide
UPK3A	aaacGCCCGCATCAAAGCGCCTAC	UPK3A Allele-specific sgRNA antisense oligonucleotide
UPK3A	accgCCCGGTGGGATTGCGCCCTG	UPK3A Locus-specific sgRNA sense oligonucleotide
UPK3A	aaacCAGGGCGCAATCCCACCGGG	UPK3A Locus-specific sgRNA antisense oligonucleotide

Table S3: List of primers used for multi-sgRNA cloning, related to STAR Methods.

Primer	Sequence
PB905	GGCTACGAAGACTATGCCCCAAACTCATCAATGTATCT
PB906	TTCTACGAAGACCCCATAAATTTACGAGCTTTCTGG
PB907	GGCTACGAAGACTATATGCCAAACTCATCAATGTATCT
PB908	TTCTACGAAGACCCAGTTAATTTACGAGCTTTCTGG
PB909	GGCTACGAAGACTAAACTCCAAACTCATCAATGTATCT
PB910	TTCTACGAAGACCCGAATAATTTACGAGCTTTCTGG
PB911	GGCTACGAAGACTAATTCCCAAACTCATCAATGTATCT
PB916	TTCTACGAAGACCCTCTGAATTTACGAGCTTTCTGG

Table S4: List of target specific primers used for amplifying bisulfite treated gDNA samples, related to STAR Methods.

Gene	Primer	Sequence
LY75 FP	NR367	ACACTCTTTCCCTACACGACGCTCTTCCGATCTNNNNNCATTGTGGTTAAGGTGATTAAGGGT
LY75 RP	NR368	GTGACTGGAGTTCAGACGTGTGCTCTTCCGATCTGATCTACCTTCCAACCTAAAACAAA
LY75 FP	NR409	ACACTCTTTCCCTACACGACGCTCTTCCGATCTNNNNNATGTGTGGTTAAGGTGATTAAGGGT
LY75 RP	NR410	GTGACTGGAGTTCAGACGTGTGCTCTTCCGATCTTATCTACCTTCCAACCTAAAACAAA
FAM181B FP	NR369	ACACTCTTTCCCTACACGACGCTCTTCCGATCTNNNNNCATGATTTTTAAAGGTTGGAATAGG
FAM181B RP	NR370	GTGACTGGAGTTCAGACGTGTGCTCTTCCGATCTGATRACAATAATAAAAAACCTACTATACC
FAM181B FP	NR411	ACACTCTTTCCCTACACGACGCTCTTCCGATCTNNNNNAGCGATTTTTAAAGGTTGGAATAGG
FAM181B RP	NR412	GTGACTGGAGTTCAGACGTGTGCTCTTCCGATCTTATRACAATAATAAAAAACCTACTATACC
UPK3A FP	NR373	ACACTCTTTCCCTACACGACGCTCTTCCGATCTNNNNNCATGTAATAGAGTTTTTATAAATAG
UPK3A RP	NR374	GTGACTGGAGTTCAGACGTGTGCTCTTCCGATCTGATAAAACACCTACCAACTCTAA
UPK3A FP	NR415	ACACTCTTTCCCTACACGACGCTCTTCCGATCTNNNNNATGGTAATAGAGTTTTTATAAATAG
UPK3A RP	NR416	GTGACTGGAGTTCAGACGTGTGCTCTTCCGATCTTATAAAACACCTACCAACTCTAA
LRRK1 FP	NR471	ACACTCTTTCCCTACACGACGCTCTTCCGATCTNNNNNCAGTTTATATGTATAAGGATGTTGGGA
LRRK1 RP	NR472	GTGACTGGAGTTCAGACGTGTGCTCTTCCGATCTTATACCCAAAAATAACATCTAAAA
RP11 FP	NR467	ACACTCTTTCCCTACACGACGCTCTTCCGATCTNNNNNCAGGGTTTTAGGAAGYGTTTTGGTGTAG
RP11 RP	NR468	GTGACTGGAGTTCAGACGTGTGCTCTTCCGATCTTATCCTTCTAATCACAAATAACT
CTBP1 FP	NR463	ACACTCTTTCCCTACACGACGCTCTTCCGATCTNNNNNCAGAATAAGAGTAGGGTTTGTAGT
CTBP1 RP	NR464	GTGACTGGAGTTCAGACGTGTGCTCTTCCGATCTTAGCTAAAATTATACAACTATCTC
SDF4 FP	NR457	ACACTCTTTCCCTACACGACGCTCTTCCGATCTNNNNNCATTGTTTTTGTGAGAAGAGGAY
SDF4 RP	NR458	GTGACTGGAGTTCAGACGTGTGCTCTTCCGATCTTATTTTCTAACTCAAAAAAAACAAC
PCDH19 FP	NR459	ACACTCTTTCCCTACACGACGCTCTTCCGATCTNNNNNCAAGTAGTTTTTTAGGTATTGTT
PCDH19 RP	NR460	GTGACTGGAGTTCAGACGTGTGCTCTTCCGATCTTATCTCCCCCTTCCTCTACTAAC
TUBB2B FP	NR461	ACACTCTTTCCCTACACGACGCTCTTCCGATCTNNNNNCATAAAGAGAGAGAGAGAGAGAGATTAA
TUBB2B RP	NR462	GTGACTGGAGTTCAGACGTGTGCTCTTCCGATCTTATACATTAAAATCRACTTAAACCCC
MEST FP	PB995	ACACTCTTTCCCTACACGACGCTCTTCCGATCTNNNNNATCGGTATTTTATTTTTGTGGTTATG
MEST RP	PB996	GTGACTGGAGTTCAGACGTGTGCTCTTCCGATCTTGAAACAACTACAACCACTC
MEST FP	PBZ10	ACACTCTTTCCCTACACGACGCTCTTCCGATCTNNNNNTACGGTATTTTATTTTTGTGGTTATG
MEST RP	PBZ11	GTGACTGGAGTTCAGACGTGTGCTCTTCCGATCTACAAACAACTACAACCACTC
SLC6A3 FP	PB648	ACACTCTTTCCCTACACGACGCTCTTCCGATCTNNNNNTCGACGAGGTTTTTTAGGTTTAGTTTT
SLC6A3 RP	PB649	GTGACTGGAGTTCAGACGTGTGCTCTTCCGATCTNNNNNCTACTCCTAAAAACTCCATTCCTCC
SLC6A3 FP	PB671	ACACTCTTTCCCTACACGACGCTCTTCCGATCTNNNNNCTGACGAGGTTTTTTAGGTTTAGTTTT
SLC6A3 RP	PB672	GTGACTGGAGTTCAGACGTGTGCTCTTCCGATCTNNNNNTACGCCCTAAAAACTCCATTCCTCC

Table S5: List of primers with Illumina adapters used in the second PCR reaction for analysis of bisulfite treated gDNA, related to STAR Methods.

Primer	Sequence
PB346	AATGATACGGCGACCACCGAGATCTACACATTACTCGACACTCTTTCCCTACACGACGCTCTTCCGATCT
PB347	AATGATACGGCGACCACCGAGATCTACACTCCGGAGAACACTCTTTCCCTACACGACGCTCTTCCGATCT
PB348	AATGATACGGCGACCACCGAGATCTACACCGCTCATTACACTCTTTCCCTACACGACGCTCTTCCGATCT
PB349	AATGATACGGCGACCACCGAGATCTACACGAGATTCCACACTCTTTCCCTACACGACGCTCTTCCGATCT
PB350	CAAGCAGAAGACGGCATACGAGATCGAGTAATGTGACTGGAGTTCAGACGTGTGCTCTTCCGATCT
PB351	CAAGCAGAAGACGGCATACGAGATTCTCCGGAGTGACTGGAGTTCAGACGTGTGCTCTTCCGATCT
PB352	CAAGCAGAAGACGGCATACGAGATAATGAGCGGTGACTGGAGTTCAGACGTGTGCTCTTCCGATCT
PB353	CAAGCAGAAGACGGCATACGAGATGGAATCTCGTGACTGGAGTTCAGACGTGTGCTCTTCCGATCT
PB370	AATGATACGGCGACCACCGAGATCTACACATTCAGAAACACTCTTTCCCTACACGACGCTCTTCCGATCT
PB371	CAAGCAGAAGACGGCATACGAGATTTCTGAATGTGACTGGAGTTCAGACGTGTGCTCTTCCGATCT
PB481	AATGATACGGCGACCACCGAGATCTACACGAATTCGTACACTCTTTCCCTACACGACGCTCTTCCGATCT
PB482	CAAGCAGAAGACGGCATACGAGATACGAATTCGTGACTGGAGTTCAGACGTGTGCTCTTCCGATCT
PB497	AATGATACGGCGACCACCGAGATCTACACCTGAAGCTACACTCTTTCCCTACACGACGCTCTTCCGATCT
PB498	CAAGCAGAAGACGGCATACGAGATAGCTTCAGGTGACTGGAGTTCAGACGTGTGCTCTTCCGATCT
PB516	AATGATACGGCGACCACCGAGATCTACACTAATGCGCACACTCTTTCCCTACACGACGCTCTTCCGATCT
PB517	CAAGCAGAAGACGGCATACGAGATGCGCATTAGTGACTGGAGTTCAGACGTGTGCTCTTCCGATCT
PB520	AATGATACGGCGACCACCGAGATCTACACCGGCTATGACACTCTTTCCCTACACGACGCTCTTCCGATCT
PB521	CAAGCAGAAGACGGCATACGAGATCATAGCCGGTGACTGGAGTTCAGACGTGTGCTCTTCCGATCT
PB611	AATGATACGGCGACCACCGAGATCTACACTCCGCGAAACACTCTTTCCCTACACGACGCTCTTCCGATCT
PB612	CAAGCAGAAGACGGCATACGAGATTTCGCGGAGTGACTGGAGTTCAGACGTGTGCTCTTCCGATCT
PB650	AATGATACGGCGACCACCGAGATCTACACTCTCGCGCACACTCTTTCCCTACACGACGCTCTTCCGATCT
PB651	CAAGCAGAAGACGGCATACGAGATGCGCGAGAGTGACTGGAGTTCAGACGTGTGCTCTTCCGATCT
PB652	AATGATACGGCGACCACCGAGATCTACACAGCGATAGACACTCTTTCCCTACACGACGCTCTTCCGATCT
PB653	CAAGCAGAAGACGGCATACGAGATCTATCGCTGTGACTGGAGTTCAGACGTGTGCTCTTCCGATCT
PB654	AATGATACGGCGACCACCGAGATCTACACTTCCTCCTACACTCTTTCCCTACACGACGCTCTTCCGATCT
PB655	CAAGCAGAAGACGGCATACGAGATAGGAGGAAGTGACTGGAGTTCAGACGTGTGCTCTTCCGATCT
PB659	AATGATACGGCGACCACCGAGATCTACACTGCTTGCTACACTCTTTCCCTACACGACGCTCTTCCGATCT
PB660	CAAGCAGAAGACGGCATACGAGATAGCAAGCAGTGACTGGAGTTCAGACGTGTGCTCTTCCGATCT
PB661	AATGATACGGCGACCACCGAGATCTACACGGTGATGAACACTCTTTCCCTACACGACGCTCTTCCGATCT
PB662	CAAGCAGAAGACGGCATACGAGATTCATCACCGTGACTGGAGTTCAGACGTGTGCTCTTCCGATCT
PB667	AATGATACGGCGACCACCGAGATCTACACAACCTACGACACTCTTTCCCTACACGACGCTCTTCCGATCT
PB668	CAAGCAGAAGACGGCATACGAGATCGTAGGTTGTGACTGGAGTTCAGACGTGTGCTCTTCCGATCT
PB673	AATGATACGGCGACCACCGAGATCTACACGGATCTGAACACTCTTTCCCTACACGACGCTCTTCCGATCT
PB674	CAAGCAGAAGACGGCATACGAGATTCAGATCCGTGACTGGAGTTCAGACGTGTGCTCTTCCGATCT
PB705	AATGATACGGCGACCACCGAGATCTACACTGATCACGACACTCTTTCCCTACACGACGCTCTTCCGATCT
PB706	CAAGCAGAAGACGGCATACGAGATCGTGATCAGTGACTGGAGTTCAGACGTGTGCTCTTCCGATCT
PB732	AATGATACGGCGACCACCGAGATCTACACAAGCGACTACACTCTTTCCCTACACGACGCTCTTCCGATCT
PB733	CAAGCAGAAGACGGCATACGAGATAGTCGCTTGTGACTGGAGTTCAGACGTGTGCTCTTCCGATCT

Table S6: List of qPCR primers used for expression analysis, related to STAR Methods. The annealing temperatures of the primer pairs are indicated.

**Gene**	**Primer**	**T_A**	**Sequence**
LY75 FP	NR473	62 °C	AATTCCTGGGATAAGGCACA
LY75 RP	NR474		CCTCATTATGGAGTTTTGTGA
FAM181B FP	NR515	64 °C	TGCAGCATCATCAGAAGGCA
FAM181B RP	NR516		GCCCTTGGTGCTGTAGAACT
SDHA FP	SDHA_FP	62 °C	TGGGAACAAGAGGGCATCTG
SDHA RP	SDHA_RP		CCACCACTGCATCAAATTCAT

Table S7: List of primers used for amplification of transcripts (PCR1), related to STAR Methods. The annealing temperatures of the primer pairs are indicated.

**Gene**	**Exon**	**Primer**	**T_A**	**Sequence**
LY75 FP	Exon 18	NR493	52 °C	ACACTCTTTCCCTACACGACGCTCTTCCGATCT NNNNNCATAACTATGAAGAAGCCGTCCTG
LY75 RP		NR494		GTGACTGGAGTTCAGACGTGTGCTCTTCCGAT CTTATTGATGGCTTTTAGTCCCACAAA
UPK3A FP	Exon 4	NR503	50 °C	ACACTCTTTCCCTACACGACGCTCTTCCGATCT NNNNNCATCCTACACGTCCGTGAACCG
UPK3A RP		NR504		GTGACTGGAGTTCAGACGTGTGCTCTTCCGAT CTTATCAGGGTGTGACAACCACCG

Table S8: List of primers used for amplification of genomic regions (PCR1), related to STAR Methods. The annealing temperatures of the primer pairs are indicated.

**Gene**	**Region**	**Primer**	**T_A**	**Sequence**
LY75 FP	sgRNA/dCas9 binding site	NR505	52 °C	ACACTCTTTCCCTACACGACGCTCTTCCGATCTNNNNNNCATGCCGAAGGAAAAGCGAGAAC
LY75 RP		NR506		GTGACTGGAGTTCAGACGTGTGCTCTTCCGATCTTATGGTGGATGTGGGAGGACAG
LY75 FP	Exon 18	NR507	50 °C	ACACTCTTTCCCTACACGACGCTCTTCCGATCTNNNNNNCATGTTTTGTGACAACCACCTC
LY75 RP		NR508		GTGACTGGAGTTCAGACGTGTGCTCTTCCGATCTTATTGGACATACTCAGATACCCG
UPK3A FP	sgRNA/dCas9 binding site	NR513	50 °C	ACACTCTTTCCCTACACGACGCTCTTCCGATCTNNNNNNCATTCCCATTGGTGAGAGACGC
UPK3A RP		NR514		GTGACTGGAGTTCAGACGTGTGCTCTTCCGATCTTATATTTGCATACGGCCCTGC
UPK3A FP	Exon 4	NR503	50 °C	ACACTCTTTCCCTACACGACGCTCTTCCGATCTNNNNNNCATCCTACACGTCCGTGAACCG
UPK3A RP		NR504		GTGACTGGAGTTCAGACGTGTGCTCTTCCGATCTTATCAGGGTGTGACAACCACCG
